# RepEnTools: an automated repeat enrichment analysis package for ChIP-seq data reveals hUHRF1 Tandem-Tudor domain enrichment in young repeats

**DOI:** 10.1186/s13100-024-00315-y

**Published:** 2024-04-03

**Authors:** Michel Choudalakis, Pavel Bashtrykov, Albert Jeltsch

**Affiliations:** Department of Biochemistry, Institute of Biochemistry and Technical Biochemistry, University of Stuttgart, Allmandring 31, 70569 Stuttgart, Germany

**Keywords:** Repeat elements, UHRF1, Chromatin modification, Repeat element analysis, Repeat element enrichment

## Abstract

**Background:**

Repeat elements (REs) play important roles for cell function in health and disease. However, RE enrichment analysis in short-read high-throughput sequencing (HTS) data, such as ChIP-seq, is a challenging task.

**Results:**

Here, we present *RepEnTools*, a software package for genome-wide RE enrichment analysis of ChIP-seq and similar chromatin pulldown experiments. Our analysis package bundles together various software with carefully chosen and validated settings to provide a complete solution for RE analysis, starting from raw input files to tabular and graphical outputs. *RepEnTools* implementations are easily accessible even with minimal IT skills (Galaxy/UNIX). To demonstrate the performance of *RepEnTools*, we analysed chromatin pulldown data by the human UHRF1 TTD protein domain and discovered enrichment of TTD binding on young primate and hominid specific polymorphic repeats (SVA, L1PA1/L1HS) overlapping known enhancers and decorated with H3K4me1-K9me2/3 modifications. We corroborated these new bioinformatic findings with experimental data by qPCR assays using newly developed primate and hominid specific qPCR assays which complement similar research tools. Finally, we analysed mouse UHRF1 ChIP-seq data with *RepEnTools* and showed that the endogenous mUHRF1 protein colocalizes with H3K4me1-H3K9me3 on promoters of REs which were silenced by UHRF1. These new data suggest a functional role for UHRF1 in silencing of REs that is mediated by TTD binding to the H3K4me1-K9me3 double mark and conserved in two mammalian species.

**Conclusions:**

*RepEnTools* improves the previously available programmes for RE enrichment analysis in chromatin pulldown studies by leveraging new tools, enhancing accessibility and adding some key functions. *RepEnTools* can analyse RE enrichment rapidly, efficiently, and accurately, providing the community with an up-to-date, reliable and accessible tool for this important type of analysis.

**Supplementary Information:**

The online version contains supplementary material available at 10.1186/s13100-024-00315-y.

## Background

**Fig. 1:**
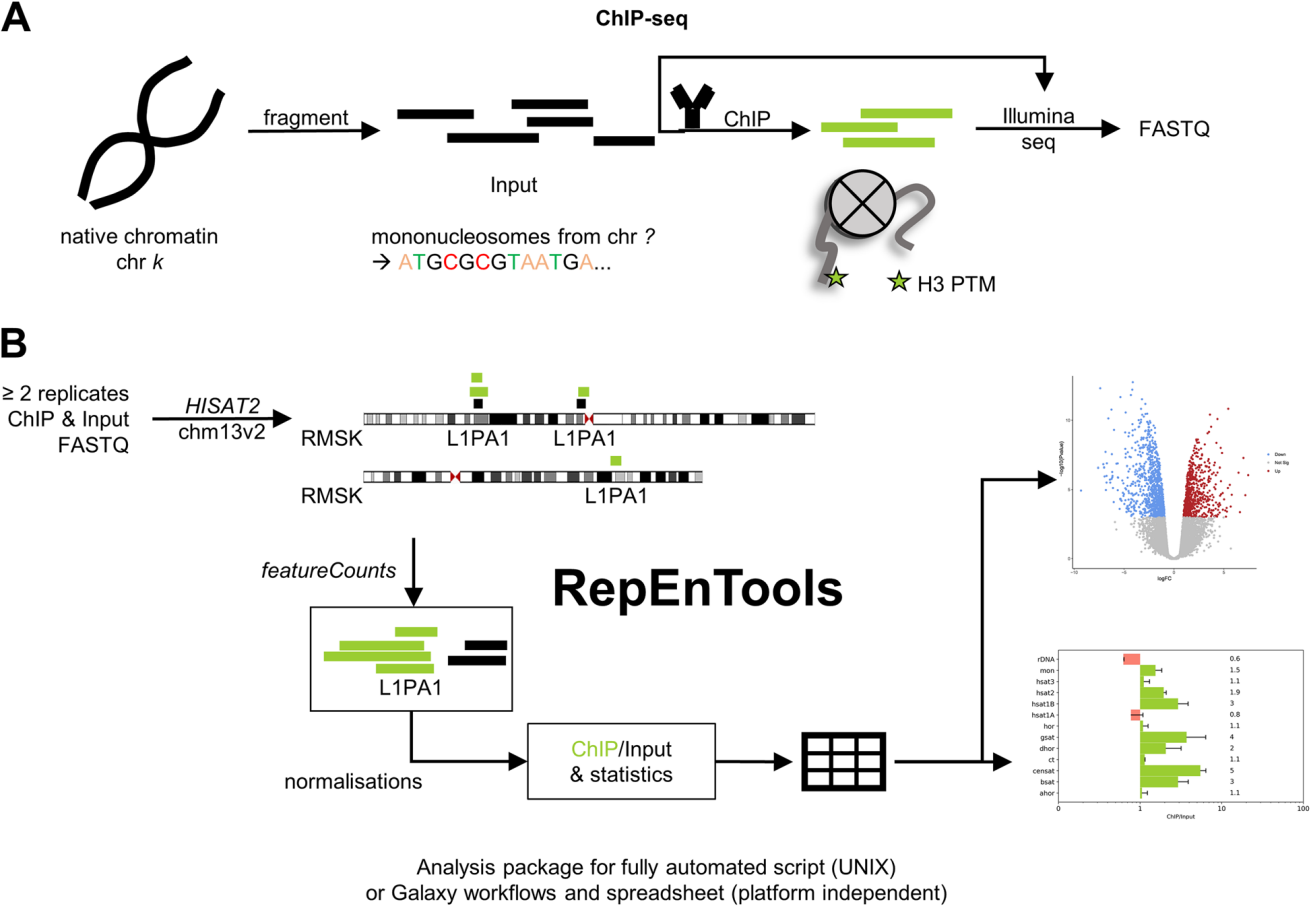
*RepEnTools* is an automated repeat enrichment analysis package for ChIP-seq data. **A** In chromatin immunoprecipitation followed by sequencing (ChIP-seq) experiments chromatin is isolated and fragmented, by sonication or MNase treatment. The nucleosomes still carry the DNA sequence information. Affinity reagents such as antibodies or chromatin interacting protein domains are then used to retain specific targets while the rest of the chromatin is washed away. The enriched DNA fragments are then isolated and sequenced, typically using the Illumina platform. As a control, an equal amount of the input chromatin is also sequenced. The FASTQ files generated contain these DNA sequences. **B** *RepEnTools* is an automated repeat enrichment analysis package for ChIP-seq data. It takes as input the FASTQ files from two replicates of ChIP-seq and the respective input chromatin datasets, trims adapters, runs QC, and aligns the sequences to chm13v2, the first complete human genome assembly. The optimised settings result in rapid and low-cost alignments, while efficiency and quality are comparable to other popular software. The repeat masker (RMSK) annotation is then used to identify and count reads on individual instances of repeat elements (REs) genome-wide, summing them up for each type of RE. The data for all experimental replicates are then collected, normalised, compared to input for enrichment or depletion, analysed for reproducibility, and reported in tables. At the end, a volcano plot of significance log_10_(*p*) versus fold-change log_2_(ChIP/input) and bar diagrams of enrichment (ChIP/input) for the RE families visualise the enrichments and the depletions of RE types

Chromatin immunoprecipitation followed by high-throughput sequencing (ChIP-seq) is a popular and very powerful technique to identify DNA sequences and the corresponding genomic loci that interact with a protein and/or carry specific post-translational modifications (PTM) of chromatin associated proteins (Fig. 1A). This experimental approach comes in many different flavours and has spawned numerous protocols, including CIDOP (Chromatin Interacting Domain Precipitation) [1]. This employs a histone modification interacting domain (HiMID) fused to an affinity tag to capture native mononucleosomes carrying a specific histone PTM. From these enriched nucleosomes, the DNA sequences are detected using short-read sequencing, typically Illumina, creating FASTQ files for bioinformatic analysis.

A principal step in the bioinformatic analysis of these experiments is matching the DNA sequences detected in the experimental pulldown samples or input (reads) to a reference genome assembly. The advent of the first gapless telomere-to-telomere (T2T) human assembly (chm13v2.0) marks the current pinnacle in linear assemblies, providing a scaffold that contains essentially all DNA sequences from a haploid hydatidiform mole and the Y-chromosome from another source [2, 3]. This led to an addition of 8% more sequences to the human reference sequence, which can have a big impact on ChIP-seq analysis of histone PTMs [4].

Analysis of typical ChIP-seq data from heterochromatin and the associated repeat elements (REs) poses many challenges, suffering from both the low relative abundance of heterochromatin in the solubilized chromatin used as starting material [5], as well as technical challenges and issues in bioinformatic processing, as their repetitive nature causes trouble in finding optimal matches for the detected sequences (reads) on the reference assembly and in assigning them to “unique” coordinates. At the same time, the assemblies do not always fully represent the underlying complexity, as shown in chm13v2.0 and the Human Pangenome [2, 3, 6, 7], with mounting evidence of sequence exchanges, duplication and evolution in certain genomic areas [6, 8, 9]. This is of particular concern for elements prone to polymorphism, such as evolutionary young repeats, centromeres, etc. which were found to vary among individual human donors [10–14]. These points demonstrate that typical ChIP-seq workflows are suboptimal to analyse such regions [4, 13–15].

In the past, a number of tools and specialised methods for RE enrichment analysis have been developed to address some of these issues [16–18], which have been described in a thorough review of the computational resources for RE analysis [19]. Additional resources can also be found on TEhub.org [20]. However, based on the current state of technology, it is possible to improve upon the previously available programmes for RE enrichment analysis by leveraging new tools, enhancing accessibility and user friendliness, and adding some key functions. To this end, we created *RepEnTools*, a RE enrichment analysis package for ChIP-seq and similar experiments. The programme is designed to use FASTQ files from two replicates of ChIP-like chromatin pulldown experiments and their respective fragmented input chromatin to trim, map, and analyse the pulldown data, and report the statistically reproducible enrichments in RE groupings (Fig. 1B). *RepEnTools* is fast, efficient and accurate, with readily comprehensible steps and bias reduction strategies. It can be implemented with minimal (UNIX version) or even zero programming skills (Galaxy workflows).

UHRF1 (Ubiquitin like with PHD And Ring Finger Domains 1) is a multidomain protein comprising a Ubiquitin-Like domain (UBL), Tandem-Tudor domain (TTD), Plant Homeodomain (PHD), SET- and RING-associated (SRA) domain, and Really Interesting New Gene (RING) domain. UHRF1 stands at the center of several epigenetic processes, including DNA methylation and gene expression regulation [21, 22]. It is a known reader of H3K9me2/3, via the TTD [23, 24], interacts with various chromatin associated proteins, and accumulates on H3K9me2/3 foci of pericentric heterochromatin of interphase nuclei [25]. Recently, we investigated genome-wide binding of hUHRF1-TTD using CIDOP-seq, and focusing on well-resolved (non-blacklisted) regions of the hg38 assembly, we demonstrated highly preferential enrichment in nucleosomes carrying H3K4me1-K9me2/3 [26]. However, this work did not address possible binding of UHRF1 in heterochromatin, as the repetitive nature and the low coverage in typical ChIP-seq data made the enrichment analysis challenging.

Here, we demonstrate that the newly developed *RepEnTools* analysis package is fast, efficient, and accurate, i.e. suitable for multi-sample repeat enrichment analysis of UHRF1 TTD CIDOP-seq data. Using *RepEnTools* we discover binding of human UHRF1-TTD in evolutionary young primate and hominid specific, polymorphic REs (SVAs and L1PA1/L1HS) and detected mouse endogenous full-length UHRF1 at young mouse specific RE loci (IAPLTRs and IAPEz). We corroborate these results experimentally by targeted CIDOP-qPCR for human TTD. With *RepEnTools*, we provide the programme package and the necessary files for implementation in human chm13v2.0 and mouse mm39 including scripts for automated installation and use (UNIX version) or as Galaxy workflows with an accompanying platform independent spreadsheet. This enables the research community to conduct high-throughput repeat element enrichment analysis of ChIP-seq data with ease and high efficiency.

## Results and discussion

### Development of *RepEnTools*

Inspired by previous works [16–18] and the latest innovations in bioinformatics and genomics, we developed *RepEnTools* aiming to further develop previously established methods for repeat element (RE) enrichment analyses (Table 1). The central idea, first presented in the seminal *Repeat Enrichment Estimator* [16], is to align HTS sequencing reads from pulldown experiments to a genome assembly with RE annotation. The annotation contains information on the specific types of REs, and their higher-order classification. Then, reads on REs are counted and summed by RE type, subfamily, family as appropriate. Finally, comparison to expected abundance reveals enrichments and depletions (Fig. 1B). Later developments provided an updated and more accessible solution for this type of analysis [17], and looked into the details that constitute best practices to analyse RE enrichment [18]. However, in recent years, significant progress in sequencing technologies and data processing have given us much improved means to conduct this type of bioinformatic research. The usage of new tools and meticulously validated details of implementation grant to *RepEnTools* unique features in RE enrichment analysis. Prime among them is the use of *HISAT2*, a graph aligner capable of handling SNPs and small InDels in an efficient manner [27], and the use of chm13v2.0, a gapless telomer-to-telomer (T2T) human genome assembly [2, 3], allowing for analysis of repeat masker (RMSK, developed by A.F.A. Smit, R. Hubley, and P. Green; see repeatmasker.org) annotated regions [13] (Table 1). Comparison to input is implemented for bias reduction, while usage of two experimental replicates allows to determine statistics on enrichment and depletion in a clear and easy to interpret manner. Finally, the entire analysis package can be comprehensively installed and executed in a UNIX environment or on public Galaxy servers followed by data analysis in Excel spreadsheets, drastically increasing accessibility of RE enrichment analyses.

**Table 1:** Overview of key features of the principal previous programmes for repeat enrichment analysis of ChIP-seq data and *RepEnTools*

	*Repeat Enrichment Estimator* [16]	*RepEnrich* [17]	Teissandier et al. [18]	*RepEnTools* (This study)
Year	2010	2014	2019	2023
Genome	hg18 & RE pseudo-genome	hg19 & RE pseudo-genome	Not reported	chm13v2.0 (T2T)
Aligner	Modified *SeqMap *& *bwa*	*Bowtie1*	*STAR*	*HISAT2*
RMSK annotation	Standard & Repbase canonical & instances	Standard & instances	Standard & LTR dictionary	Adjusted standard
Normalisation to library size	✓	✓	-	✓
Use of input	Optional	Manual option	-	✓
Replicate analysis	-	manual option	-	✓
Environment	UNIX	UNIX	UNIX	Galaxy/UNIX
Installation requirements	Source code & dependencies	Source code & individual programmes & dependencies (conda)	Individual programmes & dependencies	None on public Galaxy servers, Auto-installer for individual programmes & dependencies (UNIX)
raw FASTQ → plots	-	-	-	✓

Repeat element (RE) enrichment analysis within short-read high-throughput sequencing (HTS) data, such as ChIP-seq, is a challenging task. In the past, certain publications paved the way for RE enrichment analysis [16–18]. *RepEnTools* substantially improves upon those programmes, using the landmark chm13v2.0 (T2T) human assembly, the corresponding RMSK annotation, and an improved aligner. Normalisations, usage of input for enrichment analysis and usage of two experimental replicates for statistics on enrichment reproducibility reduce biases and put the data in meaningful context. *RepEnTools* comes as a complete analysis package that can be auto-installed in a UNIX environment or used on Galaxy servers. In either case, the only required input is to define the files to be used and the maximum fragment size for each FASTQ. The scripts incorporated in the UNIX implementation will then output all the necessary files, including tables and plots of enrichment. The Galaxy workflows we provide are supplemented by a spreadsheet to easily perform the table operations and generate the plots

**Fig. 2:**
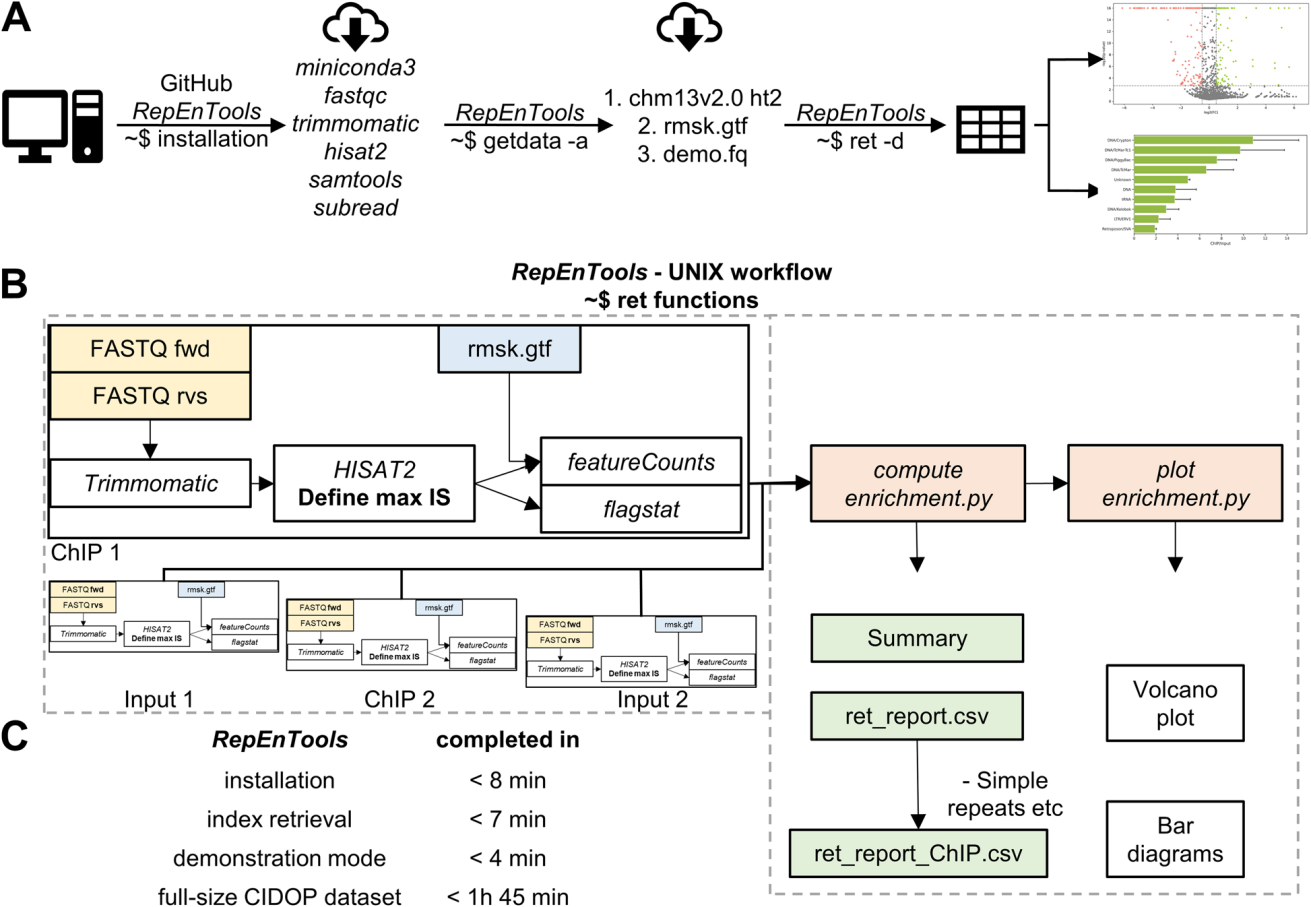
*RepEnTools* has scripts for automated installation, data analysis and plotting figures for rapid RE enrichment analysis of ChIP-seq on the human chm13v2 genome assembly. **A** *RepEnTools* in UNIX contains scripts to easily install all components, retrieve all necessary data for analysis using the human chm13v2 genome assembly and also the option to run in demonstration mode. The demo mode *RepEnTools* dataset is a reduced-size (200K reads per file) version of the UHRF1-TTD CIDOP dataset (GSE213741) providing two biological replicates. **B** Simplified workflow of the automated repeat enrichment analysis by the *RepEnTools* ~ $ ret script in UNIX. The FASTQ files (yellow) of two biological replicates of ChIP-seq and corresponding Input chromatin must be provided by the user with standardised names, and *RepEnTools* provides the repeat masker (rmsk) file for chm13v2 (blue). The white boxes indicate the data processing steps. Our Python scripts for data analysis are in light orange, and the tabular outputs are in green. Each programme is run in an individual miniconda environment, managing the changing dependencies. The ~ $ ret script regulates the conda environments, the bioinformatics packages that RepEnTools uses, the Python scripts written specifically for RepEnTools, and all subsequent steps. See also Additional file 1: Fig. S2C. **C** *RepEnTools* in UNIX is rapidly installed, and ready for use on a new UNIX system. Analysis of a full-size dataset (comprising 2 CIDOP + 2 Input FASTQ files) was consistently completed in under 105 min, using commodity hardware. Testing was conducted on a 64-bit, Intel i5-6500 @ 3.2 GHz × 4 (2015), 16 GB RAM, 1TB HDD desktop running freshly installed Ubuntu 23.04 or Debian 12.2

New UNIX users can retrieve *RepEnTools* as a zipped archive (github.com/PavelBashtrykov/RepEnTools) and use the installation commands to automatically retrieve, install and set-up all required software (Fig. 2A), followed by the necessary files for use with chm13v2. To smoothly deploy all the components of *RepEnTools* with their different dependencies, we chose the elegant solution of individual *miniconda3* environments (repo.anaconda.com/miniconda). All functions are performed by custom-made bash and python scripts. The ~ $ ret script regulates the conda environments, the bioinformatics packages that *RepEnTools* uses, the Python scripts written specifically for *RepEnTools*, and all subsequent steps. To validate the set-up, *RepEnTools* can be run in demonstration mode with reduced size datasets from two replicates.

The *RepEnTools* analysis starts with the raw FASTQ files obtained from sequencing of the ChIP-seq (or CIDOP-seq) and input libraries. These are first quality- and adapter-trimmed using *Trimmomatic*, quality controlled by *FASTQC*, and then aligned to chm13v2.0 using *HISAT2* (Fig. 2B). The optimised *HISAT2* settings (see Materials and Methods section) require an additional input of the maximum fragment size reliably observed in the specific ChIP-seq library, but a default value is provided as well (an example is shown in Additional file 1: Fig. S1). As reads from REs might be multi-mapping, meaning that they have multiple equally good matches to the reference assembly, one alignment from the group of the optimal ones is randomly assigned as the primary alignment and all others as secondary (Additional file 2: Text S1). Accurate, single counting of multi-mapping reads is achieved by the exclusive use of primary alignments in all downstream analyses. Following literature recommendations [18], *RepEnTools* does not discriminate between “unique” and multi-mapping read alignments, as final counts are anyway summed up for each type of RE. The alignments are reported as BAM files and used as input for *featureCounts* [28] along with an adjusted Repeat Masker (RMSK) gtf file used for annotation of the known REs. The latter was generated from the BED12 data available from the UCSC Table Browser [13, 29], and was adjusted to accurately reflect the correct coordinates of the REs for analysis (Additional file 1: Fig. S2A). The *featureCounts* settings were optimised to accurately represent the counts of reads (Additional file 1: Fig. S2B). The output is a table that adds up the reads that aligned to REs across the many genome-wide instances of every repeat element type.

The genome-wide and coordinate-independent tabular summary of RE counts of each ChIP-seq experiment and its matching input are then subject to normalisations. First, we normalise to the library size (the sum of mapped primary reads), effectively normalising to sequencing depth, and thereby converting the summary counts over RE types to probabilities within the specific ChIP-seq dataset. A conceptually similar approach uses probability distributions for quantitative ChIP-seq, albeit in a significantly more complex manner [30]. Next, we use the normalised counts to calculate the ChIP over input ratio and determine enrichment or depletion of specific RE groups. This step compensates for changes in experimental availability from mononucleosome generation, protocol effects including PCR amplification, as well as sequencing and software artefacts. Essentially, the input data serve as a baseline of RE representation in the starting material against which we can compare the pulldown data and determine the magnitude of the enrichment or depletion of REs in the sample pulldown. Finally, using data from two replicates, we calculate *Z*-scores and the *p*-value of enrichment or depletion, and for the summation tables of groupings (superfamilies, etc.) we normalise to the number of elements within the group. Some families contain particularly problematic elements, i.e. very short and/or elements with very low coverage. Low complexity, unknown, Simple repeats and t-RNA genes are prone to creating challenges for reliable enrichment reports. These regions are included in the analysis but removed from the bar graphs. We recommend careful evaluation of the data if their enrichment is taken into consideration. In the UNIX implementation, these calculations and operations are performed by our Python script *compute enrichment*, whereas Galaxy users can use our step-by-step instructions to generate the same output (Fig. 2B). Our *plot enrichment* script produces the two most important visualisations, while on Galaxy the corresponding tool is called *Volcano plot*. A graphical description of the *RepEnTools* workflow for Galaxy is provided in Additional file 1: Fig. S2C. On UNIX, comprehensive installation, data retrieval and demo mode were consistently completed in < 20 min. Using commodity hardware, *RepEnTools* consistently analysed a complete set of full-size datasets (comprising 2 CIDOP + 2 Input FASTQ files) in under 105 min (Fig. 2C). Step-by-step instructions and additional information for UNIX users of different skill levels can be found on github, and corresponding resources are available for Galaxy users on figshare.

### *RepEnTools *uses *HISAT2* to rapidly align reads on a T2T assembly

The alignment software, the corresponding settings and the choice of the genomic assembly can greatly affect the computational costs of the alignment, the size of the output, and its quality. Given the impact these parameters can have on RE enrichment analysis, we carefully investigated the alignment using a variety of criteria. In *RepEnTools*, we utilise *HISAT2* in optimised settings to map the trimmed, high-quality reads against the chm13v2 assembly. *HISAT2* is one of the newest mapping software available, a particularly fast and efficient de Bruijn graph aligner, able to handle SNPs and small InDels, with specialised subroutines and thereby efficiently handle repeat sequences [27]. *RepEnTools-HISAT2* represents the optimised settings of *HISAT2* used by *RepEnTools* with a defined maximum fragment length, suppression of spliced alignment, and improved randomisation of multimapping reads. In common practice, a number of aligners are used for ChIP-seq data. *STAR* is an aligner designed for RNA-seq [31], and recommended for RE enrichment analysis [18]. *Bwa-aln/backtrack* is one of the first mapping software developed for HTS [32], with some specific advantages, but largely superseded by successors. Chief among them *bwa-mem* and *bowtie2*, inarguably the two most popular mappers in the field [33, 34]. A number of pre-set expert settings are available for *bowtie2*, adjusting sensitivity and computational cost.

To reflect standardised software implementations and routine use by average users, we conducted all operations on a public Galaxy server (usegalaxy.eu). Similarly, the choice of aligners, their default settings and optimisation thereof were restricted to those available by the Galaxy GUI, and the datasets analysed were the real experimental data (*n* = 4) from a previous study [26]. These were generated from two biological replicates of ChIP-like (CIDOP) enrichment experiments and their corresponding inputs. Sequencing was performed on an Illumina NovaSeq 6000, using 150 bp paired-end (PE) reads yielding a minimum of 11 million paired-end reads for each dataset (Additional file 1: Fig. S3A).

**Fig. 3:**
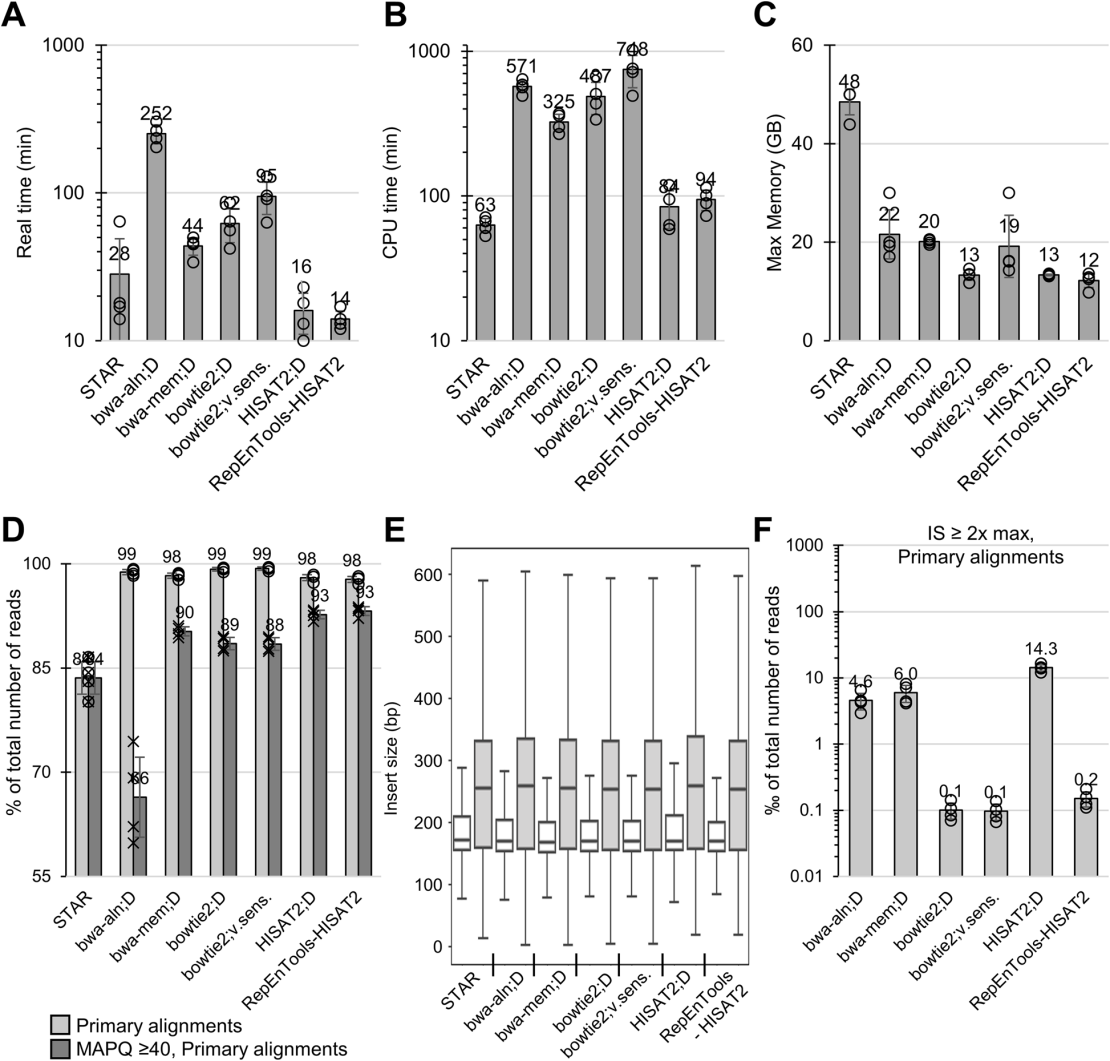
*RepEnTools* produces fast, efficient and reliable mapping of HTS reads on the human chm13v2 genome assembly. **A** *RepEnTools-HISAT2*, the alignment programme employed in *RepEnTools*, is faster than a range of popular alternatives for ChIP-seq data alignment to a T2T assembly. On average, it requires 14 min to align one ChIP-seq dataset (1.1–1.3∙10^7^ paired-end sequences). *RepEnTools-HISAT2* uses the optimised settings of *HISAT2* with a defined maximum fragment length, suppression of spliced alignment, and improved randomisation of multimapping reads. D—default. The settings are described in detail in Material and Methods. See also Additional file 1: Fig. S3A-B. The datasets (*n* = 4) originate from pulldown enriched fragments (2 datasets) and input chromatin (2 datasets). **B** *RepEnTools-*HISAT2 has low demands on CPU resources due to *HISAT2* optimised software architecture [27]. **C** *RepEnTools-HISAT2* has low memory requirements due to *HISAT2* memory minimisation strategies [27]. **D** *RepEnTools-HISAT2* generates a comparable number of primary alignments to popular alternatives using the same datasets. Application of the MAPQ ≥ 40 criterion shows comparable number of “unique, high-quality” primary alignments. See also Additional file 1: Fig. S3C. Primary mapped read counts were reported by *flagstat* (*SAMTools*) [36] for all aligners for consistency, and divided by the number of total primary reads of each BAM file. Filtering for primary reads with MAPQ ≥ 40 done using *SAMTools*. **E** All alignment algorithms produced insert sizes (IS) of comparable statistics for these very large datasets (> 10^7^ datapoints). Here, input datasets (*n* = 2) are shown beside the CIDOP datasets (*n* = 2) for each algorithm. Insert size (IS) was extracted from the TLEN of primary alignments from SAM files and plotted using *MatPlotLib*. The central line is the median, box borders are 25th to 75th percentile, and the whiskers show the deviation by 1.5 times the inter-quartile range (0.35th to 99.65th percentile in a normal distribution). **F** Using *RepEnTools-HISAT2*, the fraction of insert size (IS) outliers is comparable to the best alternatives. Among IS outliers, i.e. the inserts that exceed 2 × the length of the maximum fragment size reliably observed in the specific ChIP-seq library, discrepancies of more than an order of magnitude are seen. *STAR* alignments always have zero (0) inserts at IS ≥ 2 × max. See also Additional file 1: Fig. S3D. The data presented here were generated using the two biological replicates of hUHRF1-TTD CIDOP and their respective inputs. The bar diagrams represent the average of *n* = 4 independent datasets and the whiskers are standard deviation. Open circles show individual datapoints. All jobs were run on usegalaxy.eu, using m3.2xlarge (30 GB / 8 vCPUs / Intel Xeon E5-2670 v2 (Ivy Bridge/Sandy Bridge)) machines, except all STAR runs, that were allocated to m5d.4xlarge (64 GB / 16 vCPUs / Intel Xeon Platinum 8175) machines. All job metrics were retrieved from the individual usegalaxy.eu dataset details

To benchmark the aligner (*HISAT2*) and the optimised settings used by *RepEnTools* alongside various other popular options, we mapped real experimental reads to chm13v2 and compared computational costs. The datasets (*n* = 4) originate from pulldown enriched fragments and input chromatin, as described above. As can be seen in Fig. 3A, the *RepEnTools* settings of *HISAT2* result in faster completion of read alignment than all other software, with an average processing time of 14 min/ChIP dataset (see Material and Methods for setting details). This can be attributed to the lower CPU times that *HISAT2* required (Fig. 3B) at comparable memory requirements (Fig. 3C). *HISAT2* employs local Ferragina Manzini (FM) indexes of 57.3 kb, small enough to fit in the CPU cache rather than the slower RAM [27]. It also has optimised strategies for multithreading, and uses space efficient tables for memory minimisation. Expert users can take advantage of these to optimise the choice of resources and their allocation in high-performance computing, a particularly interesting option for large datasets or high-throughput analyses [35]. Interestingly, this efficiency directly translates to a lower financial cost of the computational operations as calculated by usegalaxy.eu (Additional file 1: Fig. S3B).

### *RepEnTools* uses *HISAT2* for efficient and reliable alignments on a T2T assembly

Having demonstrated the lower requirement of computational resources and fast processing times of the *RepEnTools-HISAT2* implementation, we wondered if this comes at the expense of quality. We retrieved the number of primary mapped reads from *SAMtools flagstat* reports [36] and compared it to primary total reads (Fig. 3D), to find comparable mapping efficiency across all aligners but *STAR*. Mapping quality (MAPQ) is an exclusion criterion that is commonly applied after mapping of ChIP-seq data despite an inconsistent and unclear implementation among different aligners. It has multiple components intending to represent the probability that a read is assigned suboptimal coordinates. Of particular note, all programmes shown here intentionally lower the score of multimapping reads, most setting it to 0 if *n* ≥ 3 regardless of alignment quality. Thus, MAPQ is ill-suited as an exclusion criterion for RE analyses. A threshold of ≥ 40 is empirically considered to reflect “unique, high-quality” alignments. This criterion is used sometimes to assess mapping quality in a dataset. Using this criterion, we observe comparable results with *RepEnTools*-*HISAT2* to the best aligners (Fig. 3D, Additional file 1: Fig. S3C). Of note, the primary output of *RepEnTools* analysis is the genome-wide and coordinate-independent tabular summary of all instances of each RE subfamily without discrimination between “unique” and multi-mapping read alignments. *RepEnTools* does not aim to analyse ChIP enrichment patterns of individual RE instances located at a specific genomic site, but only aggregated patterns of a particular RE type across the entire genome. This avoids the problems and limitations of short-read sequencing in assigning unique coordinates to RE sequences of high similarity as discussed previously in [12, 18].

An alternative indicator of the alignment quality is the insert size, i.e. the length of the sequences delimited by mapped paired reads. Finding alignments that position both reads of a pair relatively close to the experimentally expected insert size is an indication of a biologically relevant and experimentally plausible alignment. Straightforward and unambiguous, it is an established quality criterion and standard part of ChIP-seq quality control tools such as *BAMQC* [37], but seldom explicitly stated in guidelines and literature [35]. For PE reads that have one read multimapping on REs, placing both reads in relative proximity is a particularly fast way to find more biologically relevant matches, and *HISAT2* uses local FM indexes of 57.3 kb to quickly find sequence matches [27]. We separated the datasets in non-enriched input and TTD CIDOP for clarity (Fig. 3E), showing that all aligners in all settings produce insert sizes of comparable statistics. This demonstrates the generally reliable and reproducible quality of alignments with modern software.

However, as each of these plots refers to an excess of 10^7^ datapoints, we also looked at the outliers, namely the inserts that exceed 2 × the length of the maximum fragment/insert size (FS/IS) reliably observed in the specific sequencing library (Additional file 1: Fig. S1). Interestingly, a discrepancy of more than one order of magnitude is seen between aligners (Fig. 3F). With the same data, *RepEnTools*-*HISAT2* and the *bowtie2* algorithms are able to find more often solutions closer to the expected values. Of note, the < 10^4^ long inserts from *bowtie2* and *RepEnTools*-*HISAT2* in datasets that exceed 10^7^ PE sequences are a very small fraction and entirely plausible considering our definition of maximum FS, and the detection limits of such assays. While a certain number of outliers was expected, the much greater number obtained with some aligners, using the same data, is a clear red flag for the biological relevance and quality of these alignments. This behaviour was also observed for cut-off values smaller than 2 × IS.

To further illustrate the point, we counted the mapped bases of these outliers and found a staggering difference of 2 orders of magnitude. *RepEnTools*-*HISAT2* and *bowtie2* outliers originate from approximately 50 kb genome-wide, whereas *bwa-mem* outliers exceed 2,000 kb (Additional file 1: Fig. S3D), indicating that the problematic behaviour of the former is restricted to a smaller part of the genome. Hence, we interpret the reproducibly small number of outliers with *RepEnTools*-*HISAT2* as a sign of biologically more appropriate alignment and better quality. To clarify, this criterion as well as MAPQ ≥ 40 are only useful to benchmark *RepEnTools*-*HISAT2* alignment quality*. RepEnTools* analysis anyway only counts truly sequenced data, not inferred sequences between the two reads of a pair. In brief, we investigated multiple criteria to show comparable or better alignment performance with the *RepEnTools* implementation of *HISAT2* versus other popular software for a fraction of the computational cost. This validated the suitability of the *RepEnTools* aligner and its settings, as well as the genome-wide quality of the data generated by our pipeline so far.

### *RepEnTools* is suitable for analysis on RMSK regions

**Fig. 4:**
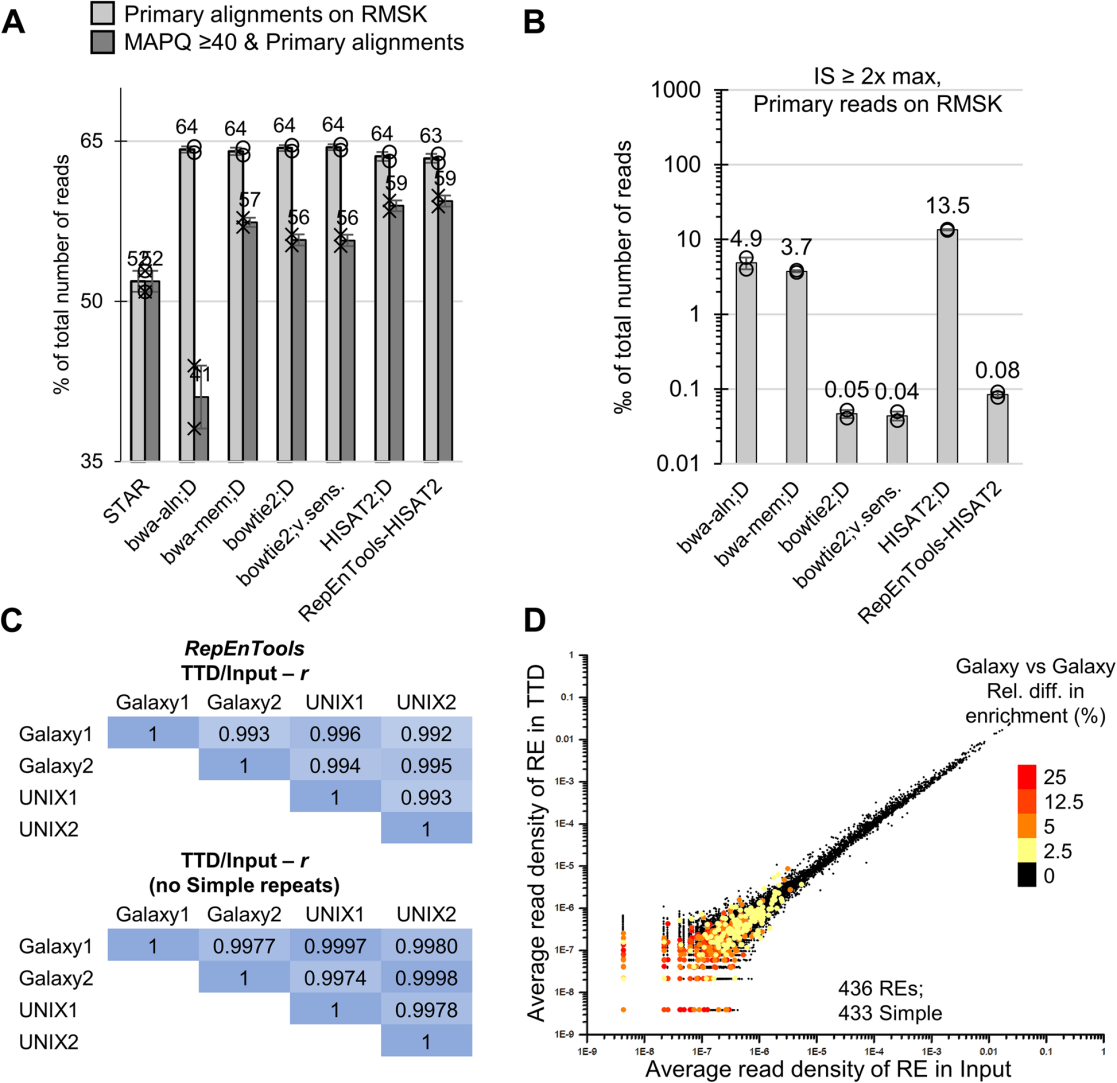
*RepEnTools* analysis is reliable for repeat masker regions, excluding some simple repeats. **A** Using real data, *RepEnTools-HISAT2* shows comparable mapping efficiency on Repeat Masker (RMSK) annotated regions to popular alternatives. Applying the MAPQ ≥ 40 criterion, we found comparable number of “unique, high-quality” primary alignments. The real data in this analysis are the two biological replicates of HepG2 inputs. CIDOP data were not used to avoid bias from experimental enrichment. The bar diagrams represent the average of *n* = 2 and the whiskers are standard deviation. D—default. Data were processed as in Fig. 3D. See also Additional file 1: Fig. S4A. **B** Using real data with *RepEnTools-HISAT2*, the fraction of insert size (IS) outliers, exceeding 2-times maximum IS, is comparable to the best alternatives. *STAR* alignments always have zero (0) inserts at IS ≥ 2 × max. Data were processed as in Fig. 3F. See also Additional file 1: Fig. S4B. **C** Using real experimental data (2 CIDOP + 2 Input), *RepEnTools* outputs enrichment scores that are well reproducible within the same implementation (Galaxy or UNIX), as well as across platforms. *RepEnTools* is even more precise when the Simple repeats are not considered. Pairwise Pearson correlations (*r*) were calculated for independent, complete runs of *RepEnTools*, considering either all 15,745 REs in RMSK, or only the 1,399 non-Simple repeat subfamilies. Each *RepEnTools* run processed all the datasets. **D** Comparison of the average enrichment scores between two complete and independent runs of the Galaxy implementation demonstrates the overall good reproduction of *RepEnTools*, while some Simple repeats are suboptimal for this type of analysis. Out of the 15,745 REs in RMSK, 436 are outliers with ≥ 2.5% relative difference in average enrichment scores. It is clear that this error in reproducibility is overwhelmingly seen among a fraction (< 500) of the 14,346 Simple repeats and correlates to low read density/abundance. See also Additional file 1: Fig. S4C-D

Some REs have significantly lower mappability than the rest of the genome [12] making them challenging targets. Having validated the suitability of *RepEnTools*-*HISAT2* in aligning experimental HTS data genome-wide, we aimed to verify the same on RMSK regions. We restricted our analysis to the aforementioned non-enriched input samples (*n* = 2) to avoid the enrichment bias of CIDOP, and benchmarked the efficiency of *RepEnTools*-*HISAT2*. Analysis of reads on RMSK regions showed that *RepEnTools*-*HISAT2* achieves comparable efficiency and number of MAPQ ≥ 40 primary alignments to the best software (Fig. 4A, Additional file 1: Fig. S4A). Looking at the number of insert size outliers, *RepEnTools*-*HISAT2* again produced comparable results to the best previously available aligners (Fig. 4B). Interestingly, *bwa-aln* and *bwa-mem* generated more large inserts than other aligners and the problem overwhelmingly stems from RMSK annotated regions (Additional file 1: Fig. S4B). *RepEnTools*-*HISAT2* and *bowtie2* produced almost two orders of magnitude more biologically relevant solutions (Additional file 1: Fig. S3D). The majority of the remaining outliers are still found on repeats, but to a smaller degree (Additional file 1: Fig. S4B).

### *RepEnTools* analyses are precise and reproducible, excluding some Simple repeats

At this point, we had validated the suitability of *RepEnTools*-*HISAT2* in aligning experimental HTS data both genome-wide and on RMSK regions, while carefully verifying the accuracy of our counting scheme with *featureCounts* (Additional file 1: Fig. S2B). After mapping and counting the sequencing data from two replicates of ChIP and input, *RepEnTools* normalises the counts to the size of the corresponding library using pseudocounts for zero reads, and calculates enrichment as ChIP over input. From the two replicates, enrichment and depletion statistics are determined using the *Z*-score and corresponding *p*-value (see Materials and Methods section). Next, we sought to validate the reproducibility of these parts of the *RepEnTools* pipeline. Using the same experimental datasets (2 CIDOP and 2 Input FASTQ files), we performed independent and complete runs of *RepEnTools* for the Galaxy and the UNIX implementation to find that *RepEnTools* RE enrichment scores are well reproducible regardless of the platform (Fig. 4C), and our programme is even more precise when the Simple repeats are not considered. The > 5 × 10^6^ RE instances in the chm13v2 human genome annotated in RMSK are grouped by their name resulting in 15,745 REs with genome-wide occurrence. To investigate reproducibility in a RE specific manner, we plotted the average read density of each RE from RMSK (*n* = 15,745) in the input data and the CIDOP data. As a third dimension, we coloured the data by relative variance across *RepEnTools* runs (Fig. 4D). Clearly, bad reproducibility correlates well with low read density in both input and CIDOP. Moreover, the REs with low read density and bad reproducibility are almost exclusively a fraction (< 500) of the 14,346 Simple repeats (Fig. 4D, Additional file 1: Fig. S4C-D).

### *RepEnTools* analysis of RMSK regions is accurate, excluding some Simple repeats

**Fig. 5:**
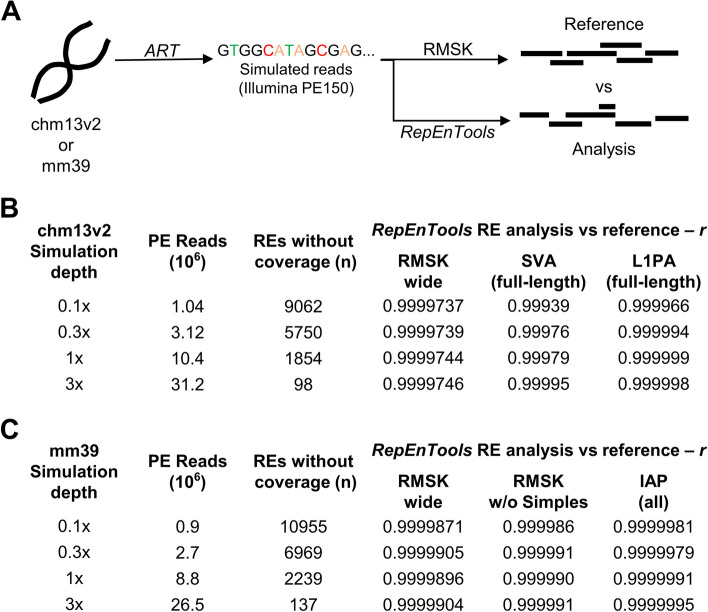
Simulated datasets show that *RepEnTools* analysis is reliable for repeat masker regions in human (chm13v2) and mouse (mm39) genomes excluding some simple repeats. **A** We used the read simulator *ART* with chm13v2 to generate simulated paired-end reads of 150 bp at various sequencing depths [38]. At the same time, *ART* created a SAM file containing the true coordinates of the simulated reads. The ground-truth data were assigned to REs while the FASTQ reads were processed by *RepEnTools*. The normalised counts from the “reference” and the *RepEnTools* analysed data were compared. This benchmarked the trimming, mapping and RMSK assignment strategies employed by *RepEnTools*. **B** Using simulated data from chm13v2, *RepEnTools* analysis of reads on RMSK annotated elements, in particular young repeats, accurately reproduces the reference data for all sequencing depths tested. At low coverage, 9,062 of the 15,745 REs in RMSK have no reads in the reference file, demonstrating the non-linear relationship between RE coverage and genome-wide coverage. See also Additional file 1: Fig. S5A-C. *RepEnTools’* analysis of reads on full-length young repeats (SVA, L1PA) is exceptionally faithful. See also Additional file 1: Fig. S5D-E. *r*—Pearson correlation. **C** *RepEnTools’* analysis using the latest mouse assembly (mm39) accurately reproduces the reference data on RMSK annotated elements for all sequencing depths tested. See also Additional file 1: Fig. S7A-B. *RepEnTools* analysis of reads on species-specific repeats (IAP) is exceptionally faithful. See also Additional file 1: Fig. S7C

To address the accuracy of the *RepEnTools* pipeline, and assess the validity of the RE assignments against data of known true origin, we employed *ART* [38], a simulator of HTS data with platform-specific artefacts. Using chm13v2 as the source of the sequences, we generated simulated data at various depths of PE reads of 150 bp using the Illumina HiSeqX TruSeq characteristics (Fig. 5A). A file containing the original coordinates of the simulated reads was also created and annotated on RMSK, named here “reference”. Then, we used the FASTQ file with simulated reads as input for *RepEnTools* and compared the analysis to the reference data. Interestingly, our simulation data substantiate the need for adequate sequencing depth in all experimental datasets, as the fingerprint plots clearly reveal the incomplete coverage for sequencing depths < 1x (Additional file 1: Fig. S5A). Inadequate genome-wide coverage adversely impacts RE analyses, as evident in the non-linear improvement of RE coverage with increasing genome-wide coverage (Fig. 5B). The high correlation between *RepEnTools* analysis and the reference conclusively verified the accuracy and suitability of the entire workflow of *RepEnTools* for RE enrichment analysis for all simulation depths tested (Fig. 5B). A closer look into the data, reveals strong correlation between REs with large errors in recovery and lower read density, as well as the propensity of Simple repeats to be challenging in analysis even in bigger datasets (Additional file 1: Fig. S5B). Exclusion of the 14,346 Simple repeats further improved the correlation between *RepEnTools* analysis and reference. The size of *RepEnTools’* error in recovering REs (excluding Simple repeats) is on average below 1% even at low coverage and improves at greater simulation depth (Additional file 1: Fig. S5C).

L1PAs, SVAs and other young REs are characterised by very low mappability [10, 12], so we investigated how well *RepEnTools* handles them, and revealed exceptional similarity to the reference. Further investigations demonstrated that *RepEnTools* analysis of these elements ranges between 99 and 100% accuracy, with a single outlier (SVA-C) at 97.8% (Additional file 1: Fig. S5D). The 1 × dataset was selected as the closest to our experimental data (Additional file 1: Fig. S3A). We also developed strategies to visualise data, which were tested on the very challenging full-length young REs (SVA, L1PA). Their suitability was validated with excellent reproducibility between reference and *RepEnTools* aligned data at a simulation depth resembling typical experimental data (Additional file 1: Fig. S5E). Taken together, our analyses demonstrate the precise, accurate, and thus reliable analysis of *RepEnTools* for REs, excluding Simple repeats. Even the notoriously challenging young REs are analysed remarkably well by *RepEnTools*. These REs are also known to cause mobile element insertions (MEI). Therefore, we investigated in a targeted manner how *RepEnTools* can handle previously identified human MEIs. These were not present in the reference assembly, and were classified to originate from ERVK, SVA, L1, and Alu [39]. The efficiency of the *RepEnTools* analysis varied by family (Additional file 1: Fig. S6). We found overall very efficient mapping (≥ 82%) and assignment to RMSK annotated regions. We expect this is due to the similarity of the MEI to the elements which are represented in chm13v2.0. Genomic deletions of REs in the samples will not affect the *RepEnTools* analysis as they are equally absent in the input chromatin and pulldown datasets.

Finally, we extended *RepEnTools* for use with the latest mouse genome (mm39). Application of the validation workflow based on simulated data prepared with *ART* [38] as described above for human chm13v2.0 revealed a high correlation between *RepEnTools* analysis and the mm39 reference (Fig. 5C), even for the mouse-specific ERVKs named IAP [40]. The size of *RepEnTools’* error in recovering murine REs (excluding Simple repeats) is on average around 1%, slightly worse than the values found for the human T2T genome. However, IAPs can be faithfully recovered (≥ 97.9%) with the recovery of potentially active IAPEz-int at 99.79% (Additional file 1: Fig. S7). Overall, these data support that *RepEnTools* can be used with additional mammalian genomes, provided a suitable RMSK file is made and the *ART* validation workflow yields convincing results.

### *RepEnTools* screening reveals hUHRF1-TTD enrichment on SVAs and ERVs

**Fig. 6:**
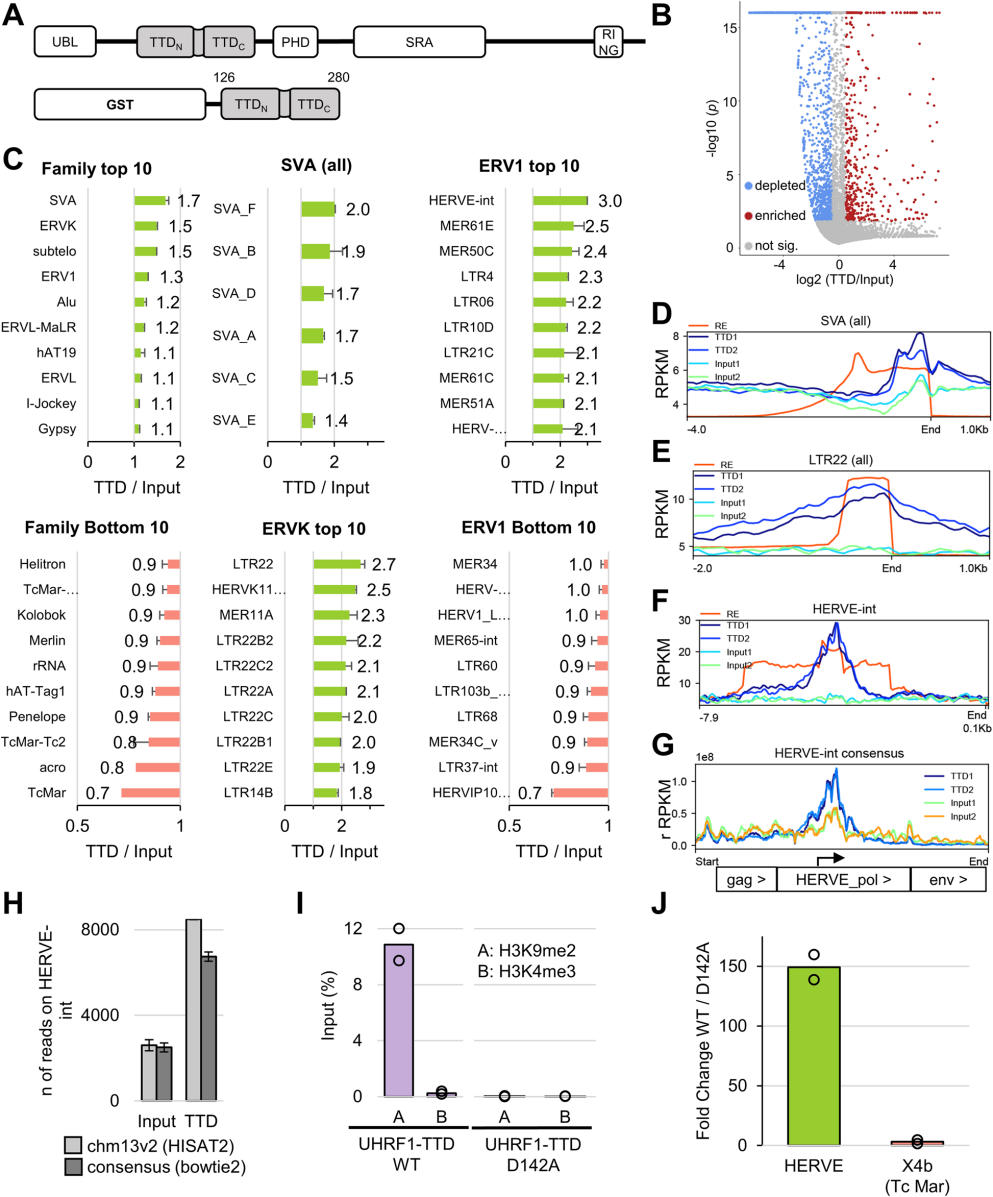
*RepEnTools* screening revealed enrichment of hUHRF1 Tandem-Tudor Domain binding on SVAs and ERVs. **A** Domain structure of the UHRF1 protein containing a Ubiquitin-Like domain (UBL), a Tandem-Tudor domain (TTD), a Plant Homeodomain (PHD), a SET- and RING-associated (SRA), and a Really Interesting New Gene (RING) domain. Bottom: Scheme of the human TTD construct (Uniprot Q96T88, residues 126–280) used in a previous work with an N-terminal GST-tag in CIDOP-seq (Chromatin Interacting DOmain Precipitation) to selectively enrich HepG2 mononucleosomes [26]. **B** The volcano plot provided by *RepEnTools* reveals the reproducible enrichment and depletion of various repeat elements (REs) by analysis of TTD CIDOP signals versus input. The plot depicts significance log_10_(*p*) versus fold-change log_2_(ChIP/input) on the y-axis and the x-axis, respectively. The colour code indicates the False Discovery Rate (FDR) adjusted *p*-values ≤ 0.05 of enrichment and depletion with statistical significance, correcting for the multiple comparisons. REs with enrichment scores of log_2_ |(TTD/Input)|≤ 0.5 are not meaningful and are coloured grey. **C** The bar diagrams from the *RepEnTools* output reveal the repeat families most and least enriched in TTD CIDOP versus input. The top genome-wide hit, the SVA family, reveals enrichment in all SVA subfamilies, but strongest in SVA-F, the youngest and hominid-specific repeat [10]. The second most enriched family, the ERVK, reveals highest enrichment in the LTR22 subfamilies. ERVK depletions can be seen in Additional file 1: Fig. S8D. Of the ERV1 family, the highest enrichment is found on HERVE-internal regions. This also represents the most enriched individual RE genome-wide. In all bar diagrams, the bar indicate the mean, and the whiskers represent the standard deviation. All data with reproducibility *p* ≤ 0.05, *n* = 2 pairs of TTD/input. **D** Localised TTD enrichment is found at the 3′ end of SVA regions, confirming *RepEnTools’* enrichment finding. Profile of all SVA models (pHMM) on chm13v2 (6274 regions), anchored to the 3′ end. RE track shows density of actual SVA annotated segments within the model. pHMM retrieved from RMSK bed12 output found on UCSC Table Browser [13, 29]. pHMM – profile Hidden Markov Model, RPKM—Reads per kilo base per million mapped reads. See also Additional file 1: Fig. S8E. For an illustration of the differences between pHMM of a RE and the actual RE see Additional file 1: Fig. S2A. **E** Broad TTD enrichment is centred on LTR22 regions, aka HERVK(HML-5) [41], confirming the enrichment findings by *RepEnTools*. Profile of all LTR22 models (781 regions). **F** Very strong TTD enrichment is seen at the center of HERVE-internal regions confirming the enrichment findings by *RepEnTools*. Profile of all HERVE-int models (142 regions). **G** TTD enrichment has a similar pattern at the center of the HERVE-int consensus (7.9 kb), when aligning the same datasets with *bowtie2*. This demonstrates that the findings of *RepEnTools* are reproducible even with a different aligner and reference. The peak in the middle of the HERVE pol gene overlaps the ORF of a 269 aa polypeptide bearing 92% similarity to a RNase H2-like domain found in reverse transcriptases. TTD and input data aligned by *bowtie2* (fast, local), consensus sequence and gene positions retrieved from dfam [42]. See also Additional file 1: Fig. S8F, Additional file 2: Text S2. **H** The alignment strategy employed in *RepEnTools* (chm13v2—*HISAT2*) aligns a comparable number of reads to an alternative strategy (consensi—*bowtie2*), for a RE well represented by its consensus sequence (HERVE-int). This reproduction validates the strategies of *RepEnTools*, and demonstrates its advantages. **I** TTD CIDOP was reproduced with WT domain and the D142A binding deficient mutant. Assayed by qPCR, a H3K9me2 reporter locus and one for H3K4me3 demonstrated similar enrichments and depletions as shown for the samples that gave the HTS data [26]. All data from *n* = 2 biological replicates, bar indicates mean. **J** The new CIDOP-qPCR experiments corroborated the TTD enrichments and depletions reported by *RepEnTools* using carefully designed and validated qPCR assays on selected targets. CIDOP-qPCR was performed using TTD and chromatin from HepG2 cells in two biological replicates. Enrichment of TTD WT over D142A represents specific over unspecific pull-down. The assays shown here target HERVE-int, the most enriched RE genome-wide, and X4b, a member of TcMar, the most depleted family. For validation of the qPCR assays see Additional file 1: Fig. S9 and S10**,** and Additional file 3: Table S2. See also Additional file 1: Fig. S10E

*RepEnTools* uses FASTQ files from two replicates of ChIP-like chromatin pulldown experiments and their respective input chromatin to produce enrichment tables, volcano plots and bar diagrams of the most relevant RE families and subfamilies. This permits rapid screening to identify enriched and depleted RE groups. To demonstrate the application of *RepEnTools*, we processed previously generated human UHRF1-TTD CIDOP-seq datasets. The analysis of hUHRF1-TTD binding on non-repetitive regions of the human genome revealed a strong association with the H3K4me1-K9me2/3 double marks [26]. Here, these datasets were analysed with *RepEnTools* to identify enrichment and depletion of RE families in the chromatin fraction bound by TTD and the new findings were validated bioinformatically as well as experimentally. The datasets were created using HepG2 micrococcal nuclease digested chromatin and hUHRF1-TTD (residues 126–280) tagged with glutathione S-transferase (GST) (Fig. 6A) [26]. Dataset sizes were described in Additional file 1: Fig. S1A. Interexperimental reproducibility of the CIDOP data was high as documented by Pearson correlation (Additional file 1: Fig. S8A). The overlapping fingerprint plots document both the reproducibility and the enrichment of CIDOP datasets versus the inputs (Additional file 1: Fig. S8B). On average, 67.7% of TTD reads were mapped to REs (Additional file 1: Fig. S8C) meaning that repeats represent a significant part of the enriched data. While this does not correspond to a strong enrichment of the 64.9% of REs in the input, the distribution of repeats is very different in pulldown and input datasets, with several families enriched or depleted in the TTD reads.

*RepEnTools* analysis revealed that many of the 15,745 REs show statistically significant genome-wide enrichments and depletions in TTD CIDOP versus input, albeit not many have a strong change (Fig. 6B). Figure 6C compiles the ten most enriched and depleted families. The most enriched RE families are SVA (SINE-R, Variable number of tandem repeats, Alu-like), a very young, primate-specific family of composite repeats (Additional file 3: Table S1). Its youngest subfamily is the highly polymorphic SVA-F [10], which is also the most enriched type of the group. The next most enriched groups are ERVKs, subtelomeric satellites (TAR) and ERV1s. Among the ERVKs, different types of LTR22 drive the trend, while others are not enriched (Additional file 1: Fig. S8D). Subtelomeric TAR loci differ in epigenetic marks from non-telomeric ones, despite insignificant sequence differences, and thus their analysis requires particular care [4]. Finally, HERVE-internal (ERV1) is the most enriched RE genome-wide (Figshare Files - Galaxy analyses). The graphical data presented here (Fig. 6B-C) are screening data generated by *RepEnTools* using the Galaxy implementation.

As primary confirmation of the output from *RepEnTools*, we focused on the enrichment calculations in our programme and investigated the groups found to contain more TTD reads than input, visualising the mapped signal profiles without additional transformations. As expected, SVA elements demonstrate localised enrichment above the input signal (Fig. 6D). A special note can be made to the very highly polymorphic sequences of VNTR in SVA, resulting in a lower input signal because of the inevitably impacted mapping (Additional file 1: Fig. S8E). As with the SVAs, profile plots of LTR22s and HERVE-int confirm the enrichments found by *RepEnTools* (Fig. 6E and F).

A second validation regarded the mapping on RE and corresponding enrichment found by *RepEnTools*. We mapped the TTD and input data against the consensus sequence of HERVE-int taken from DFAM [42] using a different aligner and reproduced the localised enrichment of TTD over the pol gene (Fig. 6G). The TTD peak on the pol gene overlaps the ORF for a 269 aa peptide that bears 92% similarity to a RNase H2-like domain found in reverse transcriptases (Additional file 1: Fig. S8F**,** Additional file 2: Text S2). This demonstrates that the findings from *RepEnTools* are reproducible even with another aligner and reference sequence. This analysis was restricted to HERVE-int as the other two top hits are not well-represented by their consensus sequence and thus suboptimal for this type of analysis. Comparison of the reads mapping to HERVE-int in chm13v2 and mapping to the HERVE-int consensus shows a 96% overlap for input and 79% overlap for TTD CIDOP (Fig. 6H). The discrepancies can be explained by the lower complexity of the consensus sequence, and hence suboptimal mapping, an apt demonstration of the advantages of the *RepEnTools* approach.

To conclusively validate our bioinformatic results, we repeated CIDOP from HepG2 chromatin with the hUHRF1-TTD WT and the binding deficient D142A mutant [26] in two biological replicates. By qPCR, we found enrichment of a H3K9me2 reporter locus and depletion of a H3K4me3 locus in the pulldown (Fig. 6I) similar to our previous report [26]. Then, we carefully developed and validated qPCR assays to evaluate the pull-down of RE loci (Additional file 1: Fig. S9 and S10, Additional file 3: Table S2). This convincingly reproduced the results obtained in our *RepEnTools* analysis, looking at the most enriched RE (HERVE-int) and a depleted RE (X4b: 0.8 TTD/Input) from the most depleted family (TcMar) (Fig. 6J).

### *RepEnTools* targeted search reveals hUHRF1-TTD enrichment on young full-length L1PAs

**Fig. 7:**
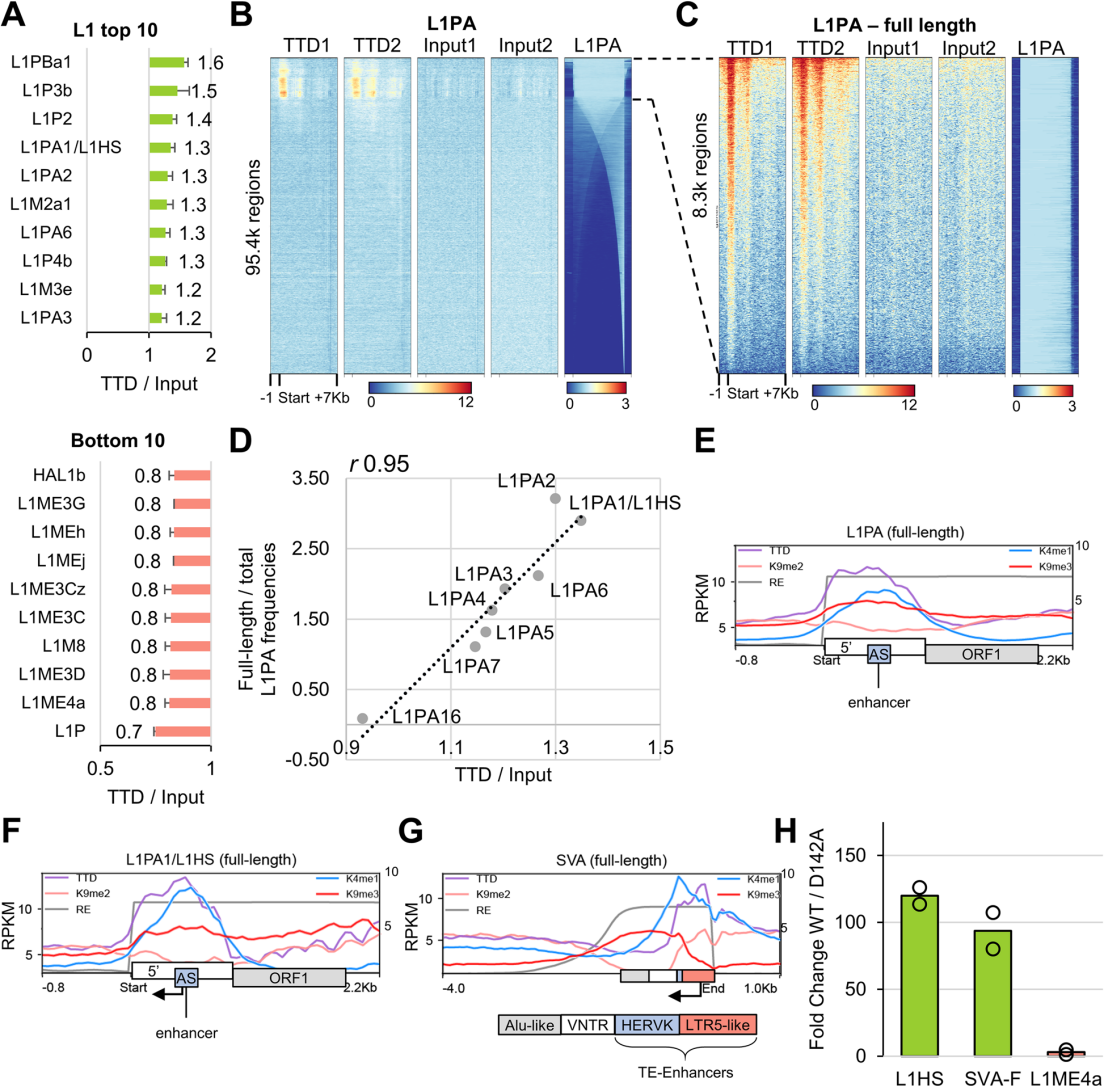
*RepEnTools* targeted search reveals enrichment of human UHRF1 Tandem-Tudor Domain binding on promoters of young, full-length L1PAs and the flanks of known enhancers. **A** The bar diagrams produced by *RepEnTools*, focusing on the L1 superfamily, reveal the subfamilies most and least enriched in TTD CIDOP versus input. The former contain the youngest evolutionary subfamilies (L1PA family), while the latter are dominated by the oldest (L1ME family). See also Additional file 1: Fig. S11A and Additional file 3: Table S1. **B** TTD enrichment is found at the 5′ end of many full-length L1Pas. This confirms *RepEnTools’* findings, and is in agreement with hUHRF1 dependent L1 promoter DNA methylation and silencing of L1 transcription [43]. Heatmap of all L1PA models (pHMM), anchored to the 5′ end, and arranged by mean L1PA track intensity. L1PA track shows position and density of actual L1PA annotated segments within the model. pHMM – profile Hidden Markov Model. See also Additional file 1: Fig. S11B. **C** Restricting analysis to the full-length L1PAs reveals strong TTD enrichment in two reproducible peaks. Heatmap arranged by mean TTD track intensity. **D** hUHRF1-TTD CIDOP enrichment correlates well with the ratio of full-length elements within L1PA subfamilies (*r* 0.95). Only reproducibly enriched/depleted subfamilies are considered (*p* ≤ 0.05, *n* = 2). Enrichment of TTD over input was calculated by *RepEnTools*, while the ratio of full-length elements is shown in Additional file 1: Fig. S11C. See also Additional file 1: Fig. S11D. **E** TTD enrichment is highest around and on the L1PA antisense promoter (AS), overlapping the H3K4me1-K9me3 double mark. This is a known enhancer, specific to the L1PA lineage, that harbours transcription factor binding motifs [11, 45]. H3K4me1 and H3K9me3 ChIP-seq RPKM scale on the right axis [46]. The vertical axes are identical for all three profile plots of this figure. The change in scale reflects the differences in experimental and sequencing methods. RE track shows density of actual L1PA annotated segments within the model. RE annotation from dfam consensus sequence [42]. See also Additional file 1: Fig. S12A. **F** TTD enrichment on the youngest subfamily of L1PAs, L1HS/L1PA1, is centred on the antisense promoter (AS), experimentally shown to strongly enhance transcription of adjacent genes [47]. RE annotation from dfam consensus sequence [42]. See also Additional file 1: Fig. S12B. **G** TTD enrichment is strongest on the HERVK and LTR5-like regions of full-length SVAs, shown to harbour TEENhancers [13, 48], overlapping the H3K4me1-K9me2/3 double marks. Annotations compiled using comparative information from dfam [42]. See also Additional file 1: Fig. S12C-E. **H** CIDOP-qPCR experiments corroborated the TTD enrichments and depletions reported by *RepEnTools* using carefully designed and validated qPCR assays on selected targets. All data from *n* = 2 biological replicates, bar indicates mean. See also Additional file 1: Fig. S10E

*RepEnTools* does not only output graphs but also generates comprehensive enrichment tables with the counts, enrichment scores and *p*-values to be analysed by user defined criteria. This can be particularly useful for a hypothesis-driven search of enrichments. Recently, shRNA mediated reduction of hUHRF1 levels was shown to result in mild loss of DNA methylation at promoters of L1 repeats and upregulation of L1-ORF1 mRNAs [43], which were shown to be transcribed from full-length young L1PA1-3/L1P1 elements [13]. Therefore, we took a closer look at hUHRF1-TTD binding at the L1 superfamily (Fig. 7A) and observed enrichment in the primate specific L1HS/L1PA1, as well as other evolutionary recent L1PAs [11, 13, 44] (Additional file 3: Table S1). First, we confirmed TTD enrichment at the 5´ end of the top hit L1PBa1 (Additional file 1: Fig. S11A). Next, we prepared a heatmap of all L1PA regions, as was done recently [13], and observed clear and reproducible enrichment of TTD on the promoter region of full-length elements (Fig. 7B). In contrast, TTD was depleted at L1ME subfamilies (Additional file 1: Fig. S11B) described as the oldest transposable elements [13, 44].

Restricting our analysis to full-length L1PAs confirmed the strong enrichment of TTD in these elements (Fig. 7C). Further analysis demonstrated that L1PA1 and L1PA2, i.e. the youngest L1s, are more often full-length than others (Additional file 1: Fig. S11C). Comparing the likelihood to observe full-length elements to the TTD/input enrichment reveals a very strong correlation of 0.95 (Fig. 7D), strongly suggesting that hUHRF1-TTD enrichment on these repeats is connected to their full-length status and hinting at a functional connection. To verify that RE abundance was not the cause of this observation, we plotted the number of L1PAs against TTD enrichment and found no correlation (Additional file 1: Fig. S11D).

To investigate a possible connection of TTD enrichment to functional elements, we summarised the data in a profile plot and annotated the functional regions present in L1PA [11, 42, 49] revealing TTD peaks at the 5′ UTR and at the intergenic region before ORF2 (Additional file 1: Fig. S11E). As a control, we looked at full-length L1MEs and no TTD enrichment was found (Additional file 1: Fig. S11F).

### hUHRF1-TTD binds to H3K4me1-K9me3 on young RE-based enhancers

Recently, we demonstrated that UHRF1-TTD, previously known as an H3K9me2/3 reader, preferentially binds to H3K4me1-K9me2 as well as H3K4me1-K9me3, but the former is far more abundant in HepG2 cells [26]. To investigate the histone marks underlying TTD peaks on L1PAs, we plotted a heatmap of the corresponding ChIP-seq data [26, 46]. This revealed a broad H3K9me2/3 density and a specific enrichment in H3K9me3 at the beginning of many full-length L1PAs. Moreover, a heatmap demonstrates the presence of the H3K4me1-K9me3 double mark in the subset of them bound strongly by TTD (Additional file 1: Fig. S12A). In addition to the previous validation of the employed visualisation workflow (Additional file 1: Fig. S5E), visualisations of *RepEnTools* alignments on representative L1PA loci show high reproducibility using biological replicates of the UHRF1-TTD CIDOP experiments as well as with simulated data (Additional file 1: Fig. S13).

To bring together TTD binding, histone marks, functional annotation and spatial information in a coherent plot, we averaged the TTD replicates and focused on the first 2 kb of the full-length L1PAs (Fig. 7E). The 5´ end of L1PAs harbours a known enhancer, specific to the L1PA lineage, bearing H3K4me1 and transcription factor binding motifs (TFBS) [11, 45]. There, the TTD peak motifs follow the H3K4me1-K9me3 double mark, as we examined in detail previously [26]. We also reproduced our recent finding of enhanced TTD binding on and around enhancers.

On the young L1PA1, TTD enrichment and the double H3K4me1-K9me3 signal was even stronger and centred on the antisense promoter (Fig. 7F**)**, experimentally shown to strongly enhance transcription of adjacent genes [47]. Intrigued by this, we extracted the TTD bound sequences and looked for TFBS using XSTREME [50] and observed enrichment of KLF4/1 and E2F4 binding motifs in both replicates **(**Additional file 1: Fig. S12B**)**, in agreement with literature data showing that KLF4 ChIP-seq was enriched in L1PA1-3 at transposable element enhancers (TE-Enhancers) [48]. These are evolutionary recent REs (L1PAs, SVAs, HERVK/LTR5HS) bearing developmental and tissue-specific enhancers [13, 48]. On SVAs these enhancers are positioned at the 3′ end. A profile plot illustrated strong H3K4me1 and TTD enrichment on the HERVK and LTR5-like regions of full-length SVAs (Fig. 7G). In line with the literature on TTD and our previous analyses [26], H3K9me2/3 was also present. Specifically, HERVK demonstrated enrichment in H3K9me3, while the LTR5-like region carried H3K9me2. A heatmap of the full-length SVAs clearly demonstrates the co-occurrence of the H3K4me1-K9me2/3 double marks and TTD binding at defined genomic loci (Additional file 1: Fig. S12C). The pattern of TTD enrichment on regions with H3K4me1-K9me3 and H3K4me1-K9me2 double marks was also found upon examination of the other top-hits of *RepEnTools* analysis, LTR22s and HERVE-int (Additional file 1: Fig. S12D-E). Interestingly, also LTR22s and HERVE-int were recently described to function as enhancers of protein-coding genes, specifically in cancers of endodermic lineage [51]. A brief analysis of HepG2 experimental literature data [52] revealed that sequences from all the REs discussed here can have enhancer functions in HepG2 cells (Additional file 1: Fig. S12F). Thus, literature reports document enhancer functions for L1PA, SVA, LTR22, HERVE in different cells.

Taken together, the histone marks, TFBS, and STARR-seq data indicate that the TEs discussed here match two recently described enhancer classes, namely the closed-chromatin enhancers and the cryptic ones [53]. These represent weakly active or inactive enhancers. Finally, we corroborated our bioinformatic findings in the wet-lab by TTD CIDOP-qPCR targeting the L1PA1/L1HS and SVA enhancer regions (Fig. 7H**,** Additional file 1: Fig. S9 and S10). The strong binding of hUHRF1-TTD to H3K4me1-K9me2/3 double marks on TE-Enhancers agrees fully with our recent report of TTD association to cell-type specific enhancers [26].

### mUHRF1 ChIP is enriched on IAPEz colocalising with H3K4me1-K9me3

**Fig. 8:**
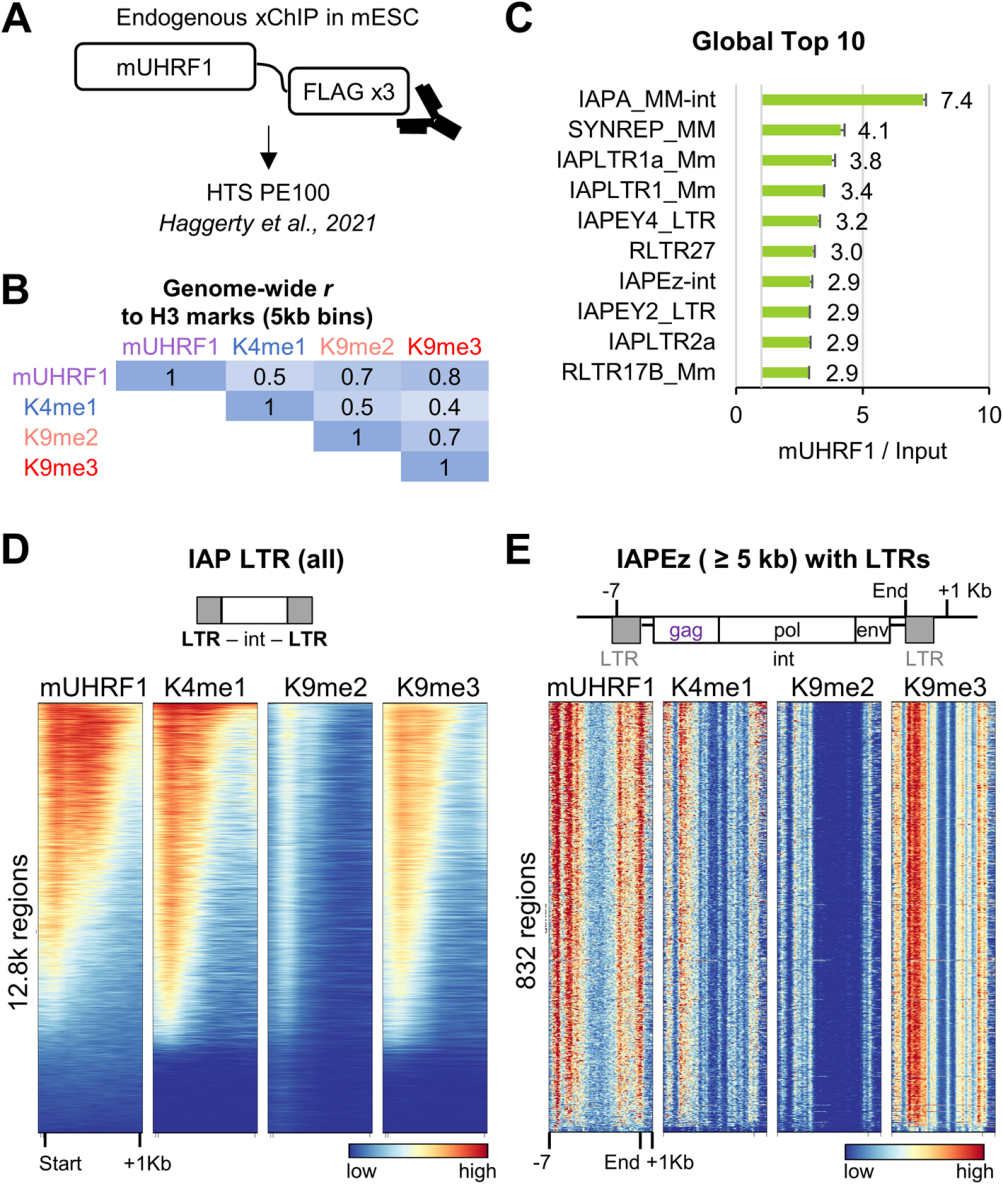
*RepEnTools* reveals enrichment of murine UHRF1 binding on young, species-specific REs with overlapping H3K4me1-K9me3. **A** Experimental design of Haggerty et al. for endogenous mUHRF1 ChIP-seq in murine ESC [54]. **B** Genome-wide Pearson correlation r-value comparison of the mUHRF1 profile to various histone H3 marks using 5 kb bins. **C** *RepEnTools* analysis reveals mUHRF1 enrichment on LTRs flanking IAP and IAPEz internal sequences. **D** Confirming *RepEnTools*’ findings, UHRF1 enrichment is found over the entire length of many IAP-LTRs at regions overlapping the H3K4me1-K9me3 double mark. IAP-LTRs contain experimentally validated enhancer sequences [57, 58]. **E** UHRF1 is enriched at the 5′ end of IAPEz longer than 5 kb, colocalising with H3K4me1- K9me3, and it was implicated in silencing IAPEz-gag expression [54, 59]. IAPEz are young, mouse-specific ERVKs. ChIP-seq data for mUHRF1 and the histone H3 marks from mESC were published previously [54, 60, 61]

ChIP-seq datasets of full-length UHRF1 are only available from mouse embryonic stem cells (mESC) [54, 55]. The technically best dataset was generated by inserting a triple FLAG-tag at the C-terminus of the endogenous UHRF1 locus in mESC. Chromatin was crosslinked, fragmented, immunoprecipitated with anti-FLAG antibody, and the DNA fragments sequenced using paired-end 100 bp for a total of 6 Gb in two replicates (Fig. 8A). Using this dataset, genome-wide comparisons of full-length UHRF1 binding to histone marks revealed good but not perfect correlation to H3K9me2 (*r* 0.7) and H3K9me3 (*r* 0.8) and some correlation to H3K4me1 (*r* 0.5) (Fig. 8B). This recapitulated the analyses of the human TTD CIDOP, shown to have similar genome-wide correlations to these marks [26]. The mESC data showed higher similarity to H3K9me3 than to H3K9me2, likely due to the drastically lower levels of H3K9me2 in ESC [56].

Application of *RepEnTools* to analyse the mUHRF1 data demonstrated enrichment in LTRs flanking IAPs, and in IAPEz, the youngest, mouse-specific subfamily of ERVK TEs (Fig. 8C). The findings of *RepEnTools* align with the literature describing IAPEz as the principal RE derepressed by UHRF1 ablation [59]. A heatmap of all IAPLTRs reveals the colocalization of H3K4me1, H3K9me3, and mUHRF1 at regions flanking the main ERV sequences (int) (Fig. 8D). The internal regions of IAPEz encode the gag, polymerase and envelope proteins of these ERVKs, and the large instances are still competent of transcription and transposition [40]. Validating these findings of *RepEnTools*, mUHRF1 enrichment is seen at the flanking LTRs and the promoter of the gag-ORF together with H3K4me1-K9me3 (Fig. 8E). Experimental reduction of UHRF1 levels in mESC has been shown to lead to IAPEz—gag upregulation, normally silenced via promoter H3K9me3 and 5mC [54, 59, 62]. As TTD is the only known element of UHRF1 able to preferentially and specifically interact with the H3K4me1-K9me2/3 containing chromatin [26], it is reasonable to associate the previously observed mUHRF1 mediated silencing of young ERVK RE, and the full-length UHRF1 and H3K4me1-K9me3 double mark enrichment presented here (Fig. 8) with the hromatin binding of the TTD domain [26]. This aligns with the hUHRF1 mediated silencing of young L1s [43] that also carry H3K4me1-K9me3 and are bound by TTD (Fig. 7). Taken together, hUHRF1-TTD or mUHRF1 and H3K4me1-K9me3 marks are enriched on the promoters of REs that are de-repressed upon UHRF1 ablation in human or mouse cells, suggesting a UHRF1-dependent silencing mechanism via TTD binding that is conserved across species, despite the absence of DNA sequence similarity between the affected REs.

### Limitations of *RepEnTools*

A persistent problem in the analysis of REs is that some REs will not be identical in reference genomes and analysed samples. This caveat is especially true for mobile elements and polymorphic sequences in short-read datasets, and can only be fully overcome by de novo RE detection and annotation, which is computationally expensive, and requires expert users [19]. *RepEnTools* applies a more accessible approach with high efficiency and low computational cost, that uses the RMSK annotation, i.e. previously annotated REs. The *RepEnTools* workflow can efficiently handle known human mobile element insertions (MEI) due to their similarity to the originating element, but cannot characterise them as MEI. Deletions of REs in the originating genomic material are not affecting the *RepEnTools* data analysis as they will appear neither in input nor in the pulldown datasets. Greatly diverging sequences will not be recognised/analysed at all.

Another general problem is assigning reads of REs to specific genomic loci, because results very much depend on the search intensity, potential variations from the reference sequence, sample sequencing mistakes and alignment parameters. Hence, it is difficult to ensure if a “unique” read is really unique and if the “best” found alignment indeed identified the true genomic origin of the read. Because of this, it can be difficult to connect RE reads from short-read datasets with specific REs at defined genomic loci [12, 18]. This problem cannot be solved by *RepEnTools* or other available standard programmes. However, the *RepEnTools* algorithm assigning multi-mapping reads randomly to one occurrence in the genome ensures a consistent, reliable and reproducible analysis of the aggregated enrichment and depletion of RE subfamilies in ChIP-seq and Pulldown-seq data as shown by the analyses presented in this manuscript.

The results generated by *RepEnTools* should be evaluated taking into consideration the limitations of the sequencing and bioinformatic processes involved, as well as the biological context of the investigated REs. We highly recommend careful wet-lab validations of key findings. We do not recomment using *RepEnTools* for TE enhancer-to-gene assignments. Any such screens should be assessed carefully and critically, cross-validated with alternative methods and confirmed in the wet-lab [40, 57].

## Conclusions

In light of the recent advances in high-throughput sequencing, the number of T2T genomes will increase in the near future. For humans there are already available a haploid (chm13v2) and most recently a diploid male T2T genome [2, 3, 7], and the human pangenome project will continue to broaden these horizons [6]. These new tools have already generated a wealth of new information and given valuable insights into some epigenetic processes on REs [4, 13, 14, 63]. At the same time, our understanding of RE function and their important influence on epigenetic regulation during development [48], in health [64, 65] and disease [66–69] increases. These developments highlight the need for better epigenome analysis tools for REs.

To address this demand, we developed *RepEnTools*, a pipeline that analyses ChIP-seq data to find enrichments of REs in a rapid and efficient manner. We benchmarked the alignment software used in *RepEnTools* and its optimized parameters to demonstrate that across multiple criteria, we produce comparable or better primary alignments compared to other popular software. Using experimental and simulated data, we establish that *RepEnTools* is suitable for reproducible and accurate RE enrichment analyses in chromatin pulldown experiments. The primary function of *RepEnTools* is to perform a genome-wide, coordinate-independent analysis summarizing data from all instances of each RE type.

To demonstrate the performance of our workflow, we used hUHRF1-TTD CIDOP data [26] and discovered TTD enrichment on young primate and hominid specific polymorphic repeats (SVA, L1PA1/L1HS), both being REs implicated in oncogenesis and development [48, 66, 68]. We corroborated the bioinformatic findings with new experimental data using carefully validated qPCR assays. The REs bound by TTD carry the H3K4me1-K9me2/3 double modification, in agreement with the preferred binding of TTD to the double marks in well-resolved genome regions discovered recently [26]. This also agrees with the hUHRF1 dependent DNA methylation and silencing of human L1 [43]. Next, in line with the previously reported TTD binding to enhancers [26], we demonstrated TTD enrichment overlaps known and putative enhancers in these repeat elements [3, 48]. Finally, we analysed mUHRF1 ChIP data to show that the endogenous murine UHRF1 protein is also found on promoters of REs colocalizing with H3K4me1 and H3K9me3. Furthermore, these RE were silenced by UHRF1 [59]. Thus, our new data suggest a functional role for UHRF1 in silencing of REs that is mediated by TTD binding to the H3K4me1-K9me3 double mark and is conserved in two mammalian species.

The implementation of *RepEnTools* has automated workflows to be user-friendly even for users without programming skills. We placed emphasis on creating a transparent, light-weight, and user-friendly analysis package that can be modularly adapted to future developments in the field, increasing longevity. *RepEnTools* will make RE analysis of ChIP-seq data more accessible, and support the community in developing further this exciting field.

## Methods

### Analysis of HTS data

Data analysis was performed on a *Galaxy* server (usegalaxy.eu) [70]. Publicly available ChIP data were obtained as raw reads from SRA (ncbi.nlm.nih.gov/sra), accession codes are given in Additional file 3: Table S3. The *SAMtools* [36], *deeptools2* [71], and *BEDtools* [72] suites, as well as *ChAsE* [73] and *Integrative Genomics Viewer* (IGV) (software.broadinstitute.org/software/igv/) [74] software were used for data processing and visualisation.

### Pre-processing of HTS data

Adapters were clipped and low-quality reads removed with *Trimmomatic* (v0.38) [75] using the default settings for PE libraries with TruSeq3 adapters, namely

-jar PE ILLUMINACLIP:$ADAPTERS_PATH/TruSeq3-PE.fa:2:30:10:8:true SLIDINGWINDOW:4:20

Output was quality controlled with *FastQC* (v0.11.8) developed by Andrews, S. (bioinformatics.babraham.ac.uk/projects/fastqc).

### Mapping of HTS data to chm13v2.0

Analyses on usegalaxy.eu used the built-in genome “Human chm13 2.0 (T2T Consortium) Jan. 2022”. The chm13v2.0.fa.gz file for UNIX was obtained from github.com/marbl/CHM13 (file created 31st March 2022). The high-quality, clean reads were mapped to chm13v2 using a variety of aligners and settings. Here we provide the settings used, as documented in the usegalaxy.eu command line. It is important to note that some settings are only seen in the command line if explicitly changed from the default values.

*STAR* (2.7.8a) [31]

*STAR* –outSAMprimaryFlag OneBestScore –outSAMmapqUnique 60 –outFilterMultimapNmax 1000 –outSAMmultNmax 1 –outFilterMismatchNmax 3 –winAnchorMultimapNmax 1000 –alignEndsType EndToEnd –alignIntronMax 1 –alignMatesGapMax 350.

The *STAR* command line is particularly verbose. Here we applied the recommended settings for RE analysis as close as possible using the Galaxy GUI [18].

*BWA* (0.7.17.5) [32]

*Bwa aln* chm13v2 && *bwa sampe* chm13v2.0 first.sai second.sai.

Here, we used the default settings of the Galaxy GUI.

*BWA-MEM* (0.7.17.2) [33]

*Bwa mem* -v 1 -I 'AverageInsertSize' chm13v2.0

Here, we used the default settings of the Galaxy GUI and the expected median insert size (range 170 – 290 bp) instead of the expected average insert length.

*Bowtie2*;def (2.5.0) [34]

*bowtie2* -p -x chm13v2.0

Here, we used the “just use defaults” pre-set settings of the Galaxy GUI.

*Bowtie2*;v.sens (2.5.0)

*bowtie2* -p -x chm13v2.0 –very-sensitive

Here, we used the “very sensitive” pre-set settings of the Galaxy GUI.

*HISAT2* (v2.2.1) [27]

*hisat2* -p -x chm13v2.0 –summary-file summary.txt

Here, we used the default settings of the Galaxy GUI. This was only included for instructive purposes. The developers of *HISAT2* recommend using the no-spliced-alignment for DNA-seq data. The effect is negligible outside the blacklisted regions on hg38, but very notable on the (peri-)centromeres of chm13v2.

*HISAT2* (v2.2.1) with *RepEnTools* settings

*hisat2* -p -x chm13v2.0 –no-spliced-alignment -I 0 -X "MaxSizefromElectropherogram" -k 5 –non-deterministic –seed '0' –summary-file summary.txt

- The no-spliced-alignment flag is required to align DNA-seq data on chm13v2, especially with an interest in genome-wide and (peri-)centromeric REs.
- The maximum fragment length for valid paired-end alignments (-X) is retrieved from experimental data (Additional file 1: Fig. S1) to allow proper insert size determination.
- Search for at most 5 (-k) distinct, primary alignments for each read is the default setting. Increasing this up to 50 resulted in minimal improvement in number of primary alignments (~ 0.1%), a testament to the strengths of this de Bruijn graph aligner.
- Using a non-deterministic pseudo-random generator for each read based on the time the read was accessed improves randomisation of primary flag assignment among multi-mapping alignments. Unexpectedly, it also reduces computational cost.

We found that deviations from these recommendations affect computational cost adversely but provide no or insignificant benefits. In the bundled version of RepEnTools we also add “–no-unal –omit-sec-seq” to suppress unaligned reads and secondary alignment quality information from the bam output, thus reducing file size.

Quality control of BAM files was done with *Qualimap 2*—*BAMQC* (v2.2.2c) [37].

### Mapping of HTS data on RE consensus sequences

RE consensus sequences used for Fig. 6G, qPCR assay design, and to identify functional regions were retrieved from dfam.org [42] as FASTA files. Alignment was done with *Bowtie2* (2.5.0) using the “fast-local” pre-set settings of the Galaxy GUI.

*bowtie2* -build 'FASTA.dat' genome && *bowtie2* -p -x 'genome' –fast-local –no-unal –omit-sec-seq

### Counting of genome-wide reads

The number of total and mapped primary reads was reported by *flagstat* (*SAMtools*) [36] for all aligners for consistency. Filtering for MAPQ ≥ 40 and exclusion of reads marked as “The alignment of this read is not primary” & “Supplementary alignment” (eq. flags 256 and 2048) was done using *SAMtools*. IS filtering was done using *BAMtools* [76].

### Statistics in figures

Standard deviations were calculated using the STDEV.P command in Excel.

### Repeat masker annotation file

The UCSC Table Browser allows retrieval of the repeat masker (RMSK) files generated specifically for chm13v2 (hub_3671779; last updated: 2022–04-27) [13, 29]. The annotation file for *RepEnTools* was generated by adjusting the bed12 file. We extracted the exons as gtf using *gene2exon1* and *bed2gff1* of the Galaxy tools, sorted and merged overlapping annotations of identical name twice (in-house script), and performed table operations to optimise the RE information for each implementation. For an illustrated example see Additional file 1: Fig. S2A. The RMSK file for mm39 (last updated: 2020–07-30) was retrieved from UCSC Table browser, inspected for correctness in IGV and optimised the RE information for *RepEnTools* using table operations.

### Quantitation of coverage on RMSK

With our adjusted RMSK gtf file (Additional file 1: Fig. S2), we employ *featureCounts* (v2.0.1) [28] to accurately count reads on the REs using the commands

*featureCounts* -F "GTF" -o "output" -s 0 -Q 0 –primary -t 'exon' -g 'gene_id' -O -M.

–minOverlap 1 –fracOverlap 0 –fracOverlapFeature 0

Average read density refers to the count of reads measured by *featureCounts* for a specific type of RE, divided by the size of the library (primary mapped reads from *flagstat*). Zero counts are replaced with 0.1 as pseudocount.

### *RepEnTools* analysis package – table operations

For each library we replace zero counts with 0.1 as pseudocount, and normalise the counts to the size of the library (primary mapped reads from *flagstat*). Then, for each RE in each biological replicate we calculate the enrichment score as ChIP/input, the arithmetic mean of two biological replicates and the standard deviation (SD). Then we calculate *Z*-scores of the enrichment:

1 $$Z=score=\frac{\left|\left(Mean\;enrichment-1\right)\right|}{\left(SD\;of\;enrichment\right)}$$

The *p*-value of each element is calculated in Excel as (1-NORM.S.DIST(Z-score,TRUE)).

In the UNIX implementation, scripts are employed to the same effect.

To generate the Volcano plot, we calculate the false discovery rate (FDR) adjusted *p*-value, using the Benjamini–Hochberg procedure [77]. First we restrict *p*-values to a minimum value of 10^–16^, then sort the 15745 REs by *p*-value (ascending), rank each as *k* = 1,…, 15745, and finally calculate the FDR-adjusted *p*-value for each element:

2 $${p}_{k}^{FDR} = {p}_{k}\cdot \frac{15745}{{\text{k}}}$$

We set the statistical significance at *α* = 0.05 level.

### *RepEnTools* UNIX package – testing

Testing was conducted on a 64-bit, Intel i5-6500 @ 3.2 GHz × 4 (2015), 16 GB RAM, 1TB HDD desktop running freshly installed Ubuntu 23.04 or Debian 12.2. The demonstration mode dataset is a reduced-size version of the UHRF1-TTD CIDOP dataset, containing just 200K reads in each file.

### Mapping of HTS data to mm39

Analyses on usegalaxy.eu used the built-in genome “mm39 Full”. *HISAT2* (v2.2.1) was used with *RepEnTools* settings, as described above.

### Simulation of HTS data

For *ART_Illumina* (V 2.5.8) [38], we employed the settings described by a very recent publication [78], only adjusting the simulation depth. This permitted the generation of data with comparable characteristics to the thorough analysis found in that publication. In brief, we selected the HiSeqX TruSeq platform profile settings, the newest Illumina settings available, and used the command

*art_illumina* -i chm13-2.fa -ss HSXt -l 150 -m 450 -f [Fold_coverage] -s 30 -p -sam -o.

with Read length: 150, Mean fragment length: 450, Std deviation: 30, Fold coverage: 0.1 to 3.

For each RE, the relative difference of average read densities between reference and *RepEnTools* analysis was

3 $$Relative\;error\;\left(\%\right)=100\cdot2\cdot\frac{\left|\left(\mathrm{Reference}-\text{Analysis}\right)\right|}{\left(\mathrm{Reference}+\text{Analysis}\right)}$$

In Additional file 1: Fig. S5D we use 100 ∙ Analysis/Reference, which is equal to 1-relative error. Pearson correlations were calculated with the PEARSON command in Excel.

### Analysis of mobile element insertion sequences using *RepEnTools*

MEI sequences were retrieved from dbRIP.org in four groups, according to attributed originating RE (ERVK, SVA, L1, and Alu) [39], reference MEIs were filtered out (column 16), then the data were converted to single .fa files with multiple entries, and used as source of genomic sequences for *ART_Illumina* (V 2.5.8) [38]. Coverage was 1x, all other settings were as described above. *RepEnTools* analysis was conducted using chm13v2.0. Each RE group was processed separately by *ART* as well as *RepEnTools*.

### Quantitation of genome-wide coverage

Using *bamcoverage* of *deepTools2* (v3.5.2), the mapped reads were quantified in 10 bp bins using Reads Per Kilobase of transcript, per Million mapped reads (RPKM) and using only primary alignments.

*bamCoverage* –outFileFormat 'bigwig' –binSize 10 –normalizeUsing RPKM –scaleFactor 1.0 –samFlagExclude 256

In Fig. 7E-G and Additional file 1: Fig. S12, biological replicates were pooled using *bigwigcompare* reporting the mean RPKM signal.

Pearson correlation factors were calculated with *multiBigwigSummary* and *plotCorrelation* (v3.5.2), using 5 kb bins for genome-wide comparisons.

*multiBigwigSummary* bins –bwfiles –binSize '5000' –distanceBetweenBins '0'

*plotCorrelation* –corData –plotFile –corMethod 'pearson' –whatToPlot 'heatmap' –colorMap 'RdYlBu_r' –plotNumbers

Fingerprint plots were created using *plotFingerprint* (v3.5.2).

*plotFingerprint* –plotFileFormat svg –outQualityMetrics –binSize '5000' –numberOfSamples '100000' –minMappingQuality '0' –samFlagExclude 256

### Heatmaps and profiles

For the bed file representing all coordinates of models for a specific RE, we started from the bed12 RMSK file retrieved from UCSC, filtered for the RE using *grep*, and retained only the first columns to create a bed6.

For the bed file representing full-length REs, we started from our adjusted RMSK gtf (Additional file 1: Fig. S2A), filtered for the RE using *grep*, filtered the regions for reciprocal ≥ 90% overlap to the model bed6 using *bedtools intersect* (v2.30.0), and converted the gtf output to bed4. Setting the overlap requirement to 100% results in many false negatives and 90% is the next possible option.

*bedtools intersect* -f 0.9 -F 0.9 -u

Heatmaps and profiles were generated using *deepTools2* (v3.5.2), with 50 bp bins

*computeMatrix* –regionsFileName 'BED.bed' –averageTypeBins 'mean' –missingDataAsZero –binSize 50

*plotHeatmap* –matrixFile –plotFileFormat 'png' –averageTypeSummaryPlot 'mean' –plotType 'lines' –missingDataColor 'black' –colorMap RdYlBu_r –alpha '1.0'

*plotProfile* –matrixFile –plotFileFormat svg –averageType 'mean'

### TFBS identifications

As the literature regarding the exact position of TEEnhancers on L1PA1/L1HS is unclear [13, 48], and the consensus sequence only contains partial motifs, we extracted the sequences from the primary alignments that overlap the full-length L1HS coordinates using *samTools-fastx* (v1.15.1), sanitised the FASTA using *filter FASTA* (v2.3), and looked for enriched TFBS motifs in the two biological replicates of UHRF1-TTD CIDOP against the corresponding inputs on meme-suite.org/meme (analysed on 10th June 2023) using *XSTREME* (v5.5.3) [50] and HOCOMOCOv11_core_HUMAN [79].

*samtools* sort -n samtools fasta -f 0 -F 2304 -G 0 input | gzip

*python* 'filter_by_fasta_ids.py' -i –min_length 15 -o

### GST-recombinant proteins

The hUHRF1-Tandem Tudor domain (UNIPROT Q96T88, residues 126–280) with a N-terminal fusion to GST, was previously purified from *E. coli*, flash-frozen and stored at -80 °C [26].

### CIDOP and ChIP

Cell culture work was performed as described previously [26]. Briefly, HepG2 cells were acquired from DSMZ—German Collection of Microorganisms and Cell Cultures (No: ACC 180) and grown in RPMI 1640 medium (Gibco) supplemented with 10% FBS, 100 U/ml penicillin and 100 mg/ml streptomycin at 37 °C under humidified air with 5% CO2. Cells were harvested at 300 g (5 min, 4 °C) and the pellets were washed once with 1 ml PBS, flash-frozen and stored at -80 °C. For CIDOP-qPCR, biological duplicates were generated.

Mononucleosome generation and CIDOP using hUHRF1-TTD wild-type and the binding deficient D142A mutant were performed as described previously [26]. Briefly, the cells were resuspended in lysis buffer (10 mM Tris–HCl pH 7.4, 2 mM MgCl2, 0.5 mM PMSF, 1 mM DTT, 0.6% v/v Igepal CA-360, EDTA-free protease inhibitor cocktail tablet), digested with ~ 135 units of MNase (NEB, M0247) per 1 million cells at 37 °C, 150 rpm for 12.5 min in one tube, diluted in interaction buffer (20 mM Tris–HCl pH 8.0, 150 mM NaCl, 1 mM PMSF, 0.1% v/v Triton X-100, 50% v/v glycerol, EDTA-free protease inhibitor cocktail tablet) centrifuged, and the supernatant containing mononucleosomes collected, flash-frozen and stored at -80 °C. One nmol of TTD was first incubated with 12 µl GST-Pierce magnetic beads for 2 h, and then with 300 nmol (60 µg) precleared chromatin for overnight binding. Beads were washed three times with PB200 (50 mM Tris–HCl pH 8.0, 200 mM NaCl, 2 mM DTT, 0.5% v/v Igepal CA-360), followed by two rinse steps (10 mM Tris–HCl pH 8.0). Samples for qPCR analysis were eluted (50 mM Tris–HCl pH 8.0, 50 mM NaCl, 5 mM EDTA, 1% w/v SDS), digested with 2.5 units of Proteinase K (NEB, P8107) at 55 °C, 900 rpm for 90 min, and purified with the ChIP DNA Purification Kit (Active Motif).

### Assessment by qPCR

All qPCR assays were performed on a CFX96 qPCR system (Bio-Rad) using ORASEE qPCR reagent (highQu) at 1 × concentration, 0.4 µl of each primer (0.267 µM final) and 1 µl of template in 15 µl final reaction volume. Master mixes were made for each primer pair used, and all samples (input for 3-point calibration, no-template control and CIDOPs) for all biological replicates were pipetted in technical triplicates, assayed on the same 96-well plate. The oligonucleotides used for qPCR assays are listed in Additional file 3: Table S2. Details on the qPCR programmes in Additional file 1: Fig. S9-S10.

### Public datasets used in this study

Published ChIP-seq data were downloaded as fastq files. Details are provided in Additional file 3: Table S3.

### Supplementary Information

**Additional file 1. **Supplementary Figures S1-10.

**Additional file 2.** Supplementary Texts S1-2.

**Additional file 3.** Supplementary Tables S1-3.

## Data Availability

Source code for the UNIX implementation of *RepEnTools* is available on github.com/PavelBashtrykov/RepEnTools and with permanent DOI on figshare.com (https://doi.org/10.6084/m9.figshare.24467956.v1). There, the workflows for the Galaxy implementation of *RepEnTools* are also available (https://doi.org/10.6084/m9.figshare.24248944.v1), along with the spreadsheet to perform table operations and generate plots, step-by-step instructions, and FAQs. Additional data relevant to the figures, including Figshare Files - Galaxy analyses that contain the tabular results of two (Galaxy1-2) runs of *RepEnTools* using hUHRF1-TTD CIDOP-HTS data, are also available on figshare (https://doi.org/10.6084/m9.figshare.24183330.v1).
