# Supplementary material for: RepEnTools: an automated repeat enrichment analysis package for ChIP-seq data reveals hUHRF1 Tandem-Tudor domain enrichment in young repeats
Source: Mob DNA. 2024 Apr 3;15:6. doi: 10.1186/s13100-024-00315-y (PMC10988844; doi:10.1186/s13100-024-00315-y)
Supplement: Supplementary file 1 — Additional file 1. Supplementary Figures S1-10. [file 13100_2024_315_MOESM1_ESM.pdf]

RepEnTools: An automated repeat enrichment analysis package for ChIP-seq data reveals hUHRF1 Tandem-Tudor domain enrichment in young repeats

Michel Choudalakis, Pavel Bashtrykov* & Albert Jeltsch*

Additional file 1

Supplementary Figures

Fig. S1. Representative example of maximum insert size determination.

Fig. S2. Reference annotation file, settings for *featureCounts*, and Galaxy workflow.

Fig. S3. Additional data regarding alignment cost and genome-wide quality.

Fig. S4. Additional data regarding alignment quality on RMSK and reproducibility of *RepEnTools*.

Figure S5. *RepEnTools* analyses REs accurately in simulated data from the human chm13v2 assembly.

Figure S6. *RepEnTools* can efficiently process known human MEIs.

Figure S7. *RepEnTools* analyses REs accurately in simulated data from the mouse mm39 assembly.

Fig. S8. Additional data related to Fig. 6.

Fig. S9. Design of qPCR assays for *RepEnTools* corroboration.

Fig. S10. Validation of qPCR assays for *RepEnTools* validation.

Fig. S11. Control data related to Fig. 7.

Fig. S12. hUHRF1-TTD binds to H3K4me1-K9me3 on functional enhancers in L1PAs.

Fig. S13. Visualisation of the reproducibility of *RepEnTools* alignments on L1PA *loci*.

Supplementary references

Supplementary Figures

Figure S1. Representative example of maximum insert size determination.

The maximum insert size (IS) is necessary information for *RepEnTools*. To demonstrate how that is determined experimentally, we present a representative capillary electropherogram of DNA isolated after chromatin pulldowns. The first peak represents DNA fragments of mononucleosome size, with dinucleosomal DNA at the second peak. The maximum IS was identified from the amplified HTS library using capillary electrophoresis (here LabChip). Bioanalyzer or similar alternatives can also be used. The maximum IS reliably observed here is ~ 570 bp – 120 bp adapters = ~ 450 bp max IS. Electropherogram generated by *LabChip GX Reviewer*.

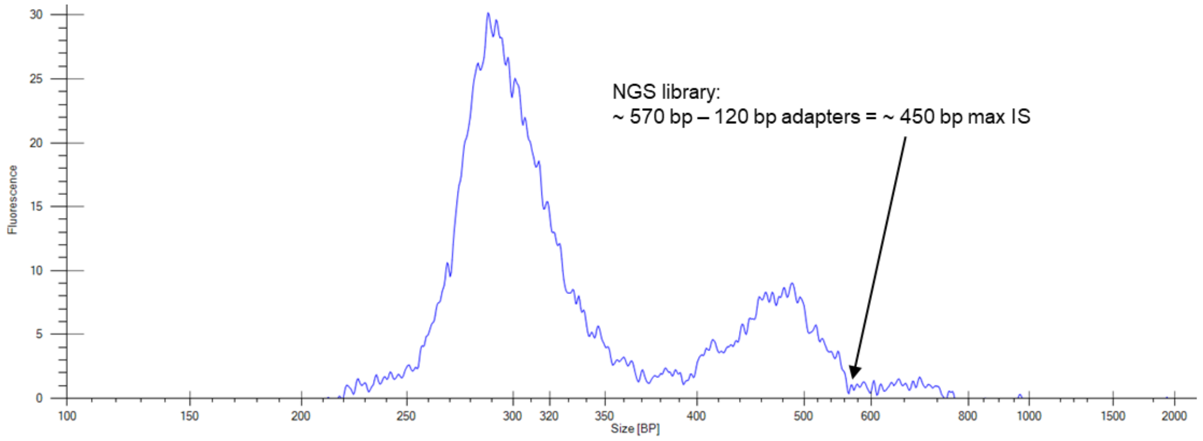

RepEnTools uses a default value of max IS = 500 bp if no other value is specified. For data without known experimental max IS this can be used as starting point and works well in many cases in our experience. To optimise the settings further a *BAMQC* report is required. In our experience, the distribution of the IS histogram typically agrees with the experimental IS distribution from capillary electrophoresis data. For reasonable max IS, this information can be used to iteratively refine the *RepEnTools* output.

Figure S2. Reference annotation file and settings for *featureCounts*.

A The repeat masker (RMSK) annotation file employed in *RepEnTools* is adjusted to correctly represent the annotated repeat element boundaries (e.g. red element). The UCSC Table Browser allows retrieval of the RMSK files generated specifically for chm13v2 (1, 2). While the BigBed file displays the elements with correct annotations, visualising the unpacked version (bed12) reveals both the pHMM model used by RMSK (thin) and the actual annotated segment (thick). Notably, the UCSC gtf output reflects only the pHMM model, not the true repeat element (vertical red dashed line). To adjust the annotation file, we extracted the exons as gtf, sorted and merged overlapping annotations of identical name twice (in-house script), and performed table operations. Browser views generated using *IGV v2.15* (*Integrative Genomics Viewer*) (3). pHMM – profile Hidden Markov Model.

B Quantification settings for *featureCounts* were selected to correctly count the tags over the annotated elements. To demonstrate, we used *featureCounts* with an RMSK file containing only the first 1000 lines, and visualised the data in *IGV*. While the first settings (**a**) are recommended in the literature (4), we employ settings (**c**) that

i. report the correct count of reads, and not any inferred sequences, representing the truly sequenced data (no -p),

ii. are invariant to the position of the second read in the pair, and are thus position and status independent (otherwise chimeric reads are counted twice, and properly paired reads once),

iii. make use of multimapping reads but only once (-primary -M),

iv. count reads that might extend over two elements, aka multi-overlapping, (-O) which is appropriate for DNA analysis,

v. are suitable for both paired- and single-end libraries.

Using only reads, instead of reads *and* inserts, allows for unambiguous normalisation to library size, and provides an essentially uniform sequence size in the same spirit as quantitative ChIP-seq (5).

C Simplified overview of the workflow using *RepEnTools* on a Galaxy server to conduct repeat enrichment analysis in ChIP-seq data. The FASTQ files (yellow) of two biological replicates of ChIP-seq and corresponding Input chromatin are provided by the user. The repeat masker (rmsk) file for chm13v2 (blue) should be retrieved from the figshare repository of *RepEnTools*. The yellow and blue boxes represent data that must be uploaded to your Galaxy library. The white boxes represent the processing steps on Galaxy. Part 1 and Part 2 are provided as separate workflows for ease of use. Analysis tables and bar plots are generated using the user-friendly spreadsheet. The workflows, gtf and spreadsheet files are available on figshare, along with step-by-step instructions. See also Fig. 2B.

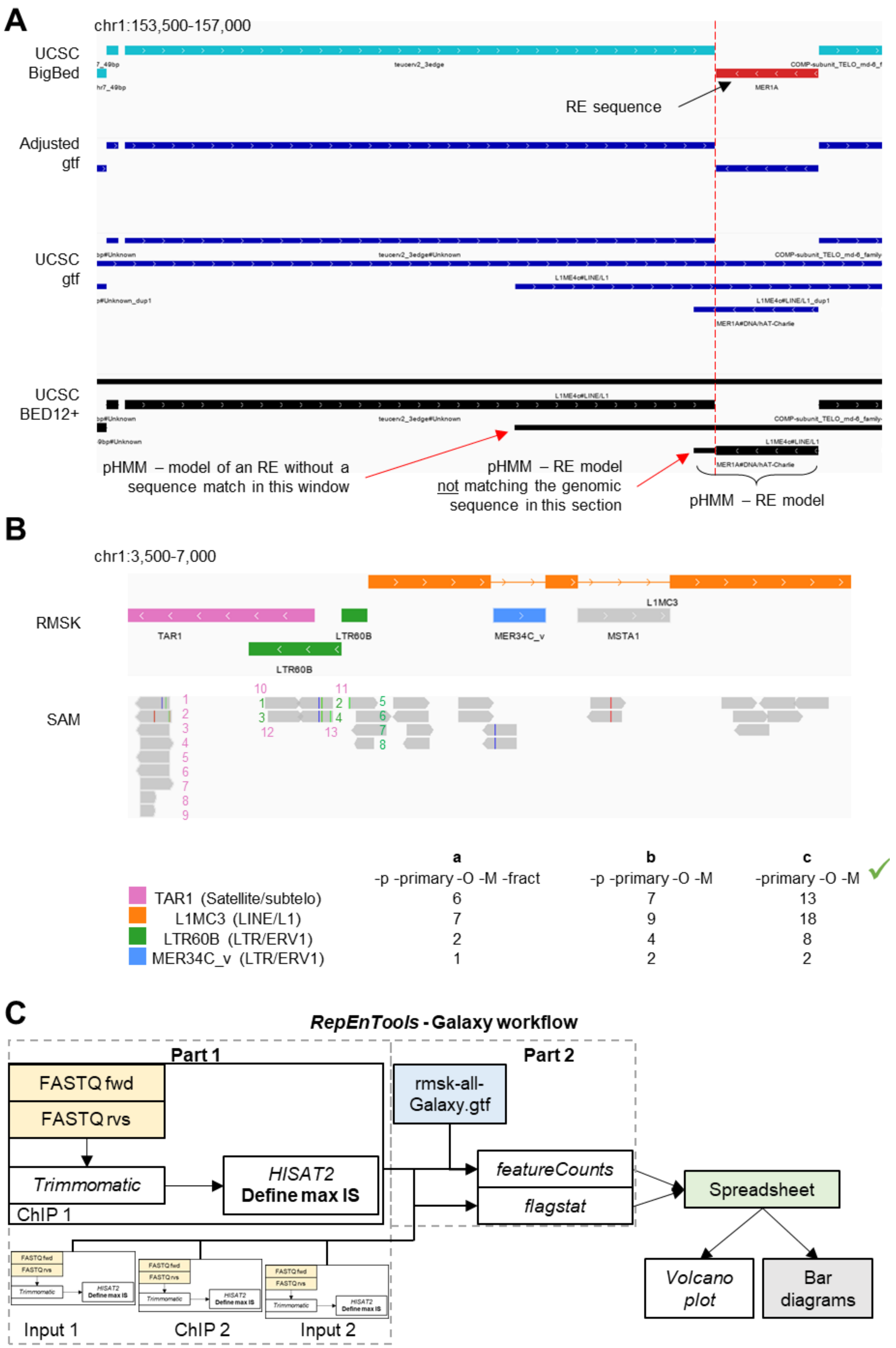
A
chr1:153,500-157,000
UCSC BigBed
Adjusted gtf
UCSC gtf
UCSC BED12+
RE sequence
pHMM – model of an RE without a sequence match in this window
pHMM – RE model not matching the genomic sequence in this section
pHMM – RE model
B
chr1:3,500-7,000
RMSK
SAM
TAR1
LTR60B
MER34C_v
MSTA1
L1MC3
a
-p -primary -O -M -fract
b
-p -primary -O -M
c
-primary -O -M
TAR1 (Satellite/subtelo) 6 7 13
L1MC3 (LINE/L1) 7 9 18
LTR60B (LTR/ERV1) 2 4 8
MER34C_v (LTR/ERV1) 1 2 2
C
RepEnTools - Galaxy workflow
Part 1
Part 2
FASTQ fwd
FASTQ rvs
Trimmomatic
HISAT2
Define max IS
ChIP 1
rmsk-all-Galaxy.gtf
featureCounts
flagstat
Spreadsheet
Volcano plot
Bar diagrams
Input 1
ChIP 2
Input 2

Figure S3. Additional data regarding alignment cost and genome-wide quality.

A Total number of primary paired-end (PE) reads within each FASTQ file analysed here exceeds 10 million. The two biological replicates of hUHRF1-TTD CIDOP and their corresponding MNase digested inputs were sequenced on an Illumina NovaSeq 6000, using 150 bp PE reads. Primary read counts were retrieved from *flagstat* (*SAMTools*).

B *RepEnTools-HISAT2* is the most cost-efficient aligner with an average cost of 0.15 US dollars to align one ChIP-seq dataset (1.1-1.3·10^7 paired-end sequences). Job cost estimates retrieved from the individual usegalaxy.eu dataset details. See also Fig. 3A-C. Open circles show individual datapoints. The datasets (n = 4) originate from pulldown enriched fragments (n = 2) and input chromatin (n = 2).

C A very high percentage of the *RepEnTools-HISAT2* alignments meet or exceed the MAPQ ≥ 40 criterion. After filtering for mapped primary, MAPQ ≥ 40 reads, read counts were retrieved from *flagstat* (*SAMTools*) and divided by the mapped primary read counts in each dataset. See also Fig. 3D.

D Using *RepEnTools-HISAT2*, the number of mapped bases from insert size (IS) outliers, PE reads that exceed the 2x maximum, is comparable to the best alternatives. This demonstrates that the problematic read alignments are restricted to smaller parts of the genome. *STAR* alignments always have zero (0) inserts at IS ≥ 2x max. Aligned data (BAM) were filtered by IS using *BamTools* and counted using *BAMQC*. See also Fig. 3F.

The data presented here were generated using the two biological replicates of hUHRF1-TTD CIDOP and their respective inputs. The bar diagrams represent the average of n = 4 and the whiskers are standard deviation. Open circles show individual datapoints. All jobs were run on usegalaxy.eu, using m3.2xlarge (30 GB / 8 vCPUs / Intel Xeon E5-2670 v2 (Ivy Bridge/Sandy Bridge)) machines, except all *STAR* runs, that were allocated to m5d.4xlarge (64 GB / 16 vCPUs / Intel Xeon Platinum 8175) machines.

A

Sample	TTD1	TTD2	Input1	Input2
n of PE reads	13.40·10^6	13.1·10^6	11.02·10^6	13.13·10^6

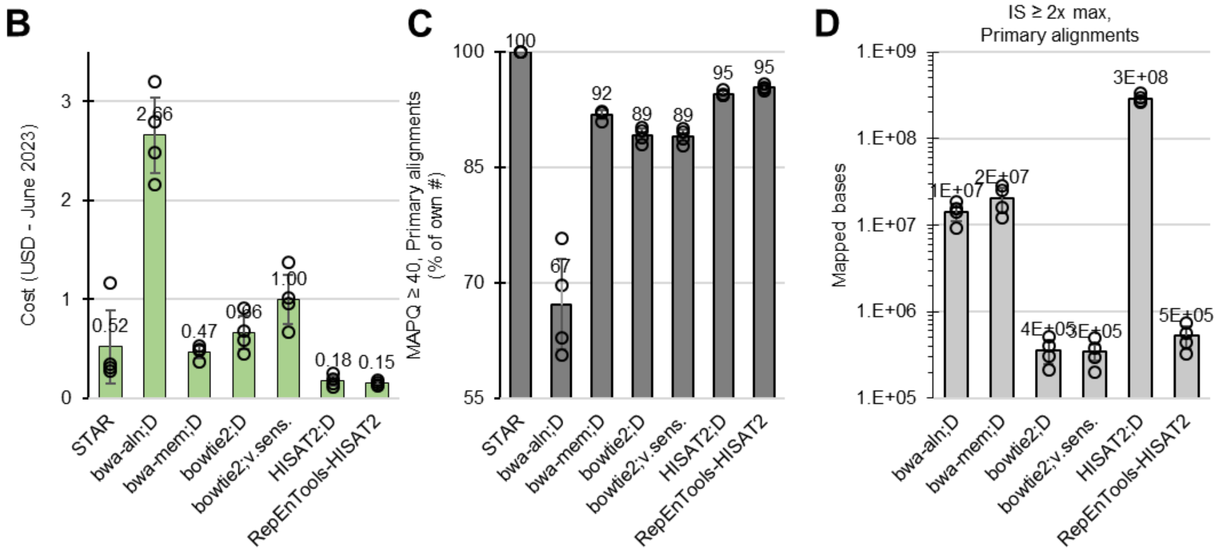

Figure S4. Additional data regarding alignment quality on RMSK and reproducibility of *RepEnTools*.

A A very high percentage of the *RepEnTools-HISAT2* alignments on RMSK (repeat masker) annotated regions meet or exceed the MAPQ ≥ 40 criterion. Data were processed as in Fig. S3C and summarily counted using *featureCounts* and the RMSK annotation. See also Fig. 4A.

B Using *RepEnTools-HISAT2*, the fraction of insert size (IS) outliers, exceeding 2 times maximum IS, originates mostly from RMSK annotated regions. *STAR* alignments always have zero (0) inserts at IS ≥ 2x max. Data were processed as in Fig. S3D and summarily counted using *featureCounts* and the RMSK annotation. See also Fig. 4B. The data presented here were generated using the two biological replicates of inputs.

The bar diagrams in panel A and B represent the average of n = 2 and the whiskers are standard deviation. Open circles show individual datapoints.

C The different implementations of *RepEnTools* result in reproducible enrichment scores using real experimental datasets. Comparison of the average enrichment scores between two complete and independent runs on UNIX demonstrates the high reproducibility of *RepEnTools,* while some Simple repeats are suboptimal for these analyses. Out of the 15,745 REs in RMSK, 430 are outliers with >2.5% relative difference in average enrichment scores. It is clear that this error in reproducibility is overwhelmingly seen among Simple repeats and correlates to low read density/abundance. For these analyses we used the TTD-CIDOP data sets (n = 2 sets of CIDOP + 2 Input). Each *RepEnTools* run processed all the datasets. See also Fig. 4D.

D Comparison of the average enrichment scores between the Galaxy and the UNIX implementations demonstrates the good inter-platform reproduction of *RepEnTools*. Of the 450 reproducibility outliers, almost all are Simple repeats with low read density/abundance.

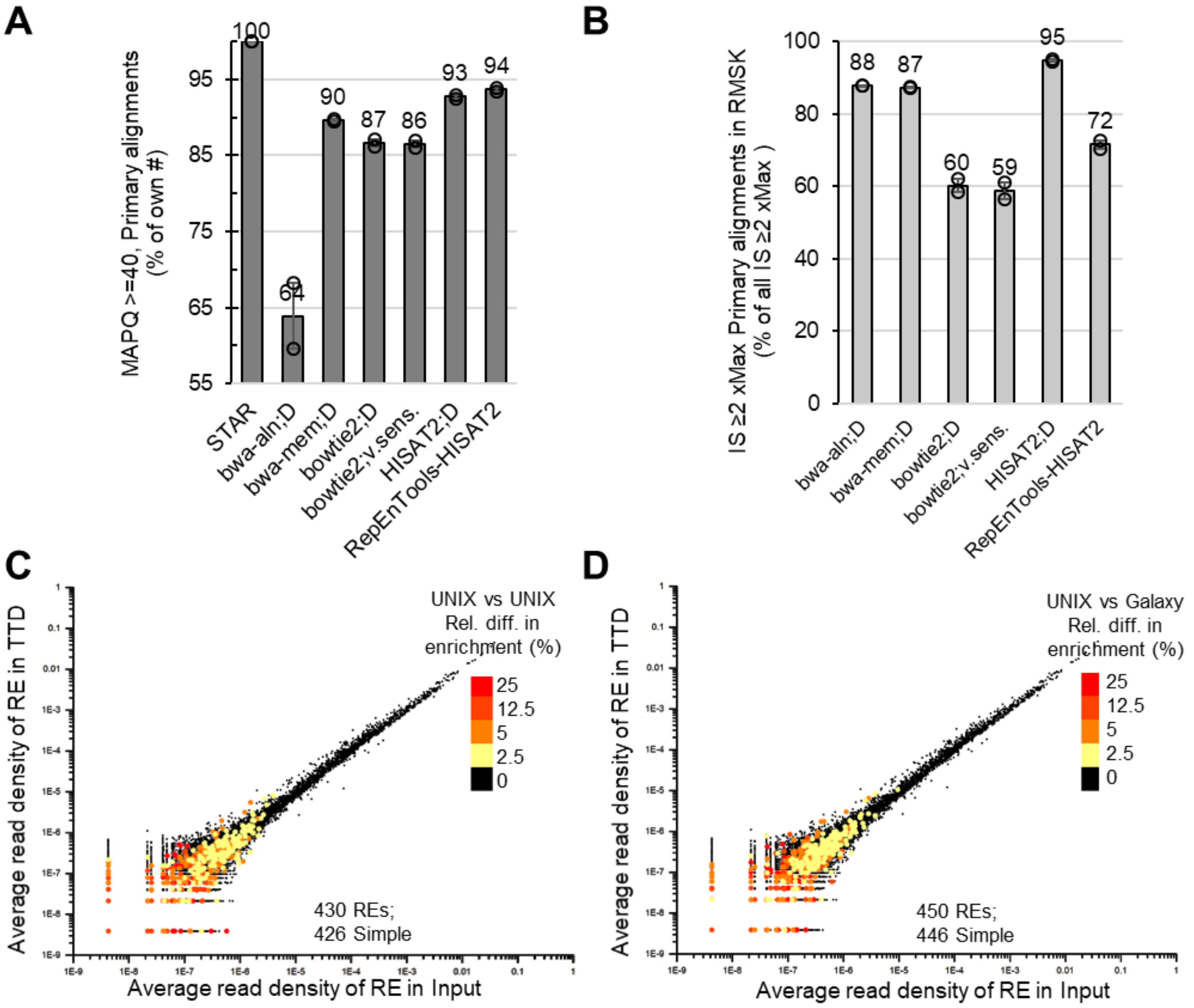

A
MAPQ >=40, Primary alignments (% of own #)
100
64
90
87
86
93
94
STAR
bwa-aln;D
bwa-mem;D
bowtie2;D
bowtie2;v.sens.
HISAT2;D
RepEnTools-HISAT2
B
IS ≥2 xMax Primary alignments in RMSK (% of all IS ≥2 xMax)
88
87
60
59
95
72
C
UNIX vs UNIX Rel. diff. in enrichment (%)
25
12.5
5
2.5
0
430 REs; 426 Simple
Average read density of RE in TTD
Average read density of RE in Input
D
UNIX vs Galaxy Rel. diff. in enrichment (%)
450 REs; 446 Simple

Figure S5. *RepEnTools* analyses REs accurately in simulated data from the human chm13v2 assembly.

A Fingerprint plots demonstrate the incomplete coverage in the simulated data at depths < 1x versus the theoretical perfect input at complete coverage (black line). This effectively mimics experimental enrichment, and underpins the necessity of adequate coverage in both ChIP and Input experimental data for meaningful analyses. To explain fingerprint plots simply, genome-wide bins overlap reads, the reads in each bin are counted, the bins are ranked by the sum of reads, and the cumulative partial sums of the reads are expressed as percentage of the whole (y-axis). The x-axis represents the percentage of bins (6). Plot generated with *plotFingerprint* (*deepTools2*) (6). See also Fig. 5B.

B *RepEnTools* demonstrates excellent accuracy in analysis of REs in all sequencing depths tested, excluding Simple repeats. Comparison of the read density between the "ground truth" reference dataset and the one analysed by *RepEnTools* demonstrates the strength of the mapping and RMSK assignment strategies used in *RepEnTools*. Out of the 15,745 REs in RMSK, the ones with the lowest read density (first quartile) are essentially exclusively some of the 14,346 Simple repeats. Other low abundance REs deviate from the diagonal, flaring slightly at bottom right. See also Fig. 5B.

C *RepEnTools* accurately analyses REs, excluding the 14,346 Simple repeats, with average relative errors below 1%, even at low coverage. Simple repeats are excluded from this analysis as a significant fraction is not present in the "ground truth" reference, skewing results (Fig. 5B). Each bar represents the average of n = 1399 REs, whiskers are the standard error of mean.

D *RepEnTools* demonstrates very accurate data recovery for the notoriously challenging full-length young REs (SVA, L1PA) at a simulation depth resembling typical real experimental data. Comparison of the read counts found on full-length young REs in the "ground truth" reference reads and the data recovered by *RepEnTools* reveals rates exceeding 97.8% for all elements. The 1x dataset was selected as the closest to our experimental data (Fig. S3A). For the box plots, the central lines show the median, box borders are 25th to 75th percentile, and whiskers are 5th to 95th percentile. See also Fig. 5B.

E The strategies used to visualise data on full-length young REs (SVA, L1PA) are appropriate for the task and with very good recovery, at a simulation depth resembling typical experimental data. Profile plots constructed similarly to those found in Fig. 6-7.

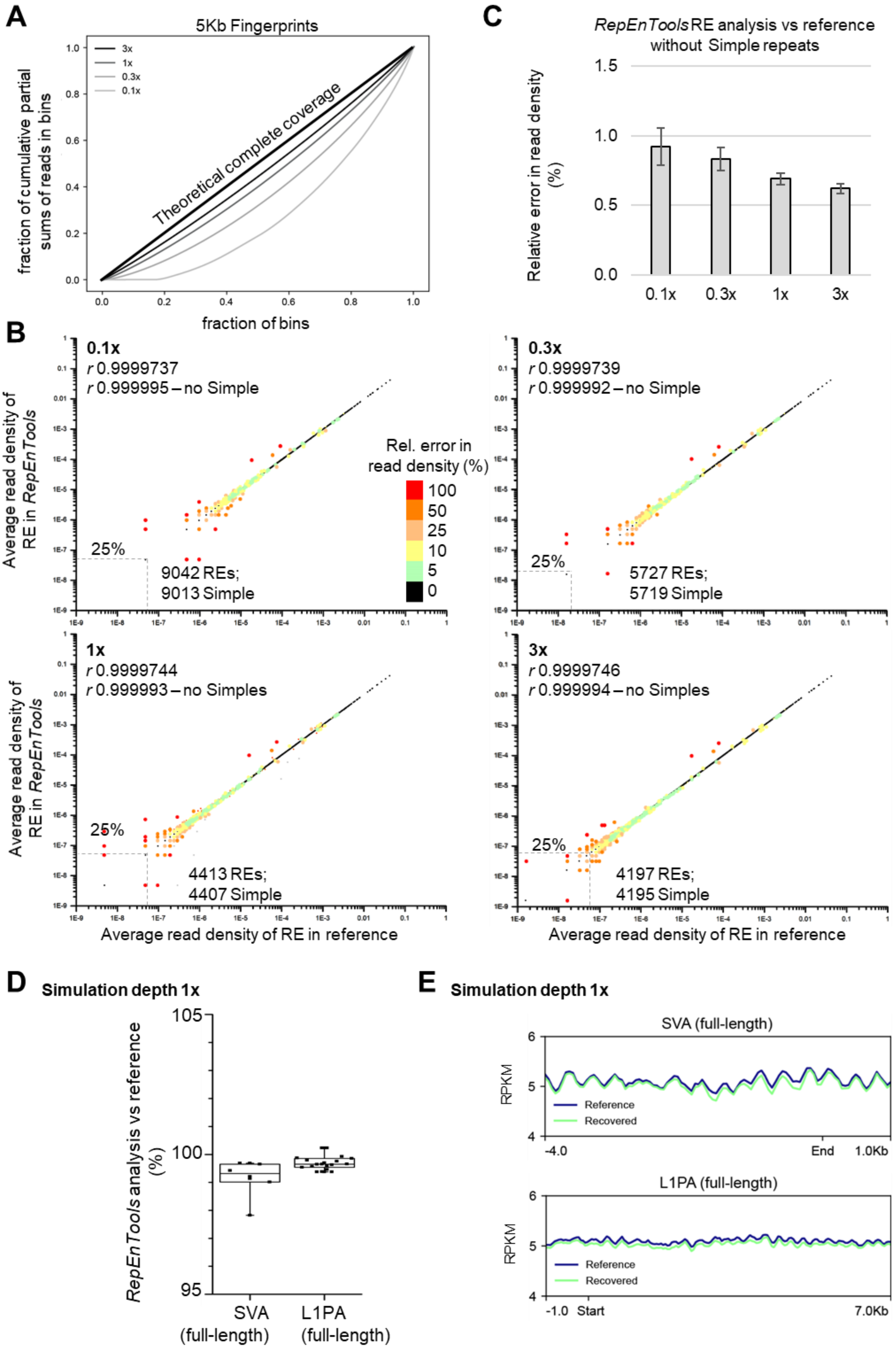
A
5Kb Fingerprints
3x
1x
0.3x
0.1x
Theoretical complete coverage
fraction of cumulative partial sums of reads in bins
fraction of bins
C
RepEnTools RE analysis vs reference without Simple repeats
Relative error in read density (%)
0.1x
0.3x
1x
3x
B
0.1x
r 0.9999737
r 0.999995 – no Simple
9042 REs; 9013 Simple
Rel. error in read density (%)
100
50
25
10
5
0
0.3x
r 0.9999739
r 0.999992 – no Simple
5727 REs; 5719 Simple
1x
r 0.9999744
r 0.999993 – no Simples
4413 REs; 4407 Simple
3x
r 0.9999746
r 0.999994 – no Simples
4197 REs; 4195 Simple
25%
Average read density of RE in RepEnTools
Average read density of RE in reference
D
Simulation depth 1x
RepEnTools analysis vs reference (%)
SVA (full-length)
L1PA (full-length)
E
Simulation depth 1x
SVA (full-length)
RPKM
Reference
Recovered
-4.0
End
1.0Kb
L1PA (full-length)
-1.0
Start
7.0Kb

Figure S6. *RepEnTools* can efficiently process known human MEIs.

A We obtained non-reference Mobile Element Insertion (MEI) sequences from the literature (dbrip.org) (7). The sequences contain the detected human MEI and 400 bp on each flank. They were converted to Illumina PE150 reads using *ART*, and processed using *RepEnTools*.

B Analysis of these MEIs using *RepEnTools* demonstrates efficient mapping and overall appropriate placement on RMSK annotated regions. This is possible because of the similarity of the transposed sequences to elements already existing in the reference, and the superior handling of SNPs and small InDels by HISAT2. Autonomous TEs (HERVK, L1) are processed more efficiently than the non-autonomous ones (SVA, Alu). Simulated PE sequences from autonomous TEs are overwhelmingly assigned to the same RE family as annotated in the database, as expected by their transposition process. MEIs are assigned to RE groups in the dbrip.org database, but sequences may contain additional REs as well as non-RE sequences.

C Control data for the simulated read datasets and summary data regarding the alignments. The Alu group has the highest number and percentage of InDels, likely contributing to the lower mapping efficiency seen in panel B. Alignment quality reports generated by *BAMQC*.

D Exemplary control data for the retrieved MEIs investigated using dfam.org. The sequences found in group L1 mostly contain very young L1PAs. Other RE types are rarely seen in those sequences, in line with the results in panel B. The sequences found in group Alu contain additional REs and a notable fraction of sequences that was not assigned to any REs. This likely accounts for the lower percentage of Alu alignments on RMSK seen in panel B.

The sequences analysed here contain MEIs and 400 bp from each flank. This resulted in fragments that covered only the inserted sequence, as well as “junction” fragments that extended over both the genomic locus of insertion and the inserted sequence. We reasoned that, while fragments from the former should have almost perfect alignments in highly similar active TEs, “junction” fragments would either have unassignable reads (SE, PE) or appear as chimeric alignments (PE only). Therefore, reads from longer MEI were expected to have higher alignment efficiency, as they would align to the TEs that are included in the reference assembly and are highly similar to their common “ancestral”/“original” sequence source. Interestingly, these expectations were reproduced in our data analysis (HERVK *vs.* L1 *vs.* Alu).

This analysis shows that *RepEnTools* can efficiently handle reads from RE sequences that are not part of the reference assembly. The principal output of *RepEnTools* is the genome-wide, coordinate-independent summation from all instances of each RE type. The efficient mapping and assignment to RMSK annotated elements we demonstrate here ensures their appropriate representation in the summation tables and enrichment scores. Assignment of specific coordinates to novel insertions has limitations when using short-read sequencing technologies and should be treated with the appropriate caution.

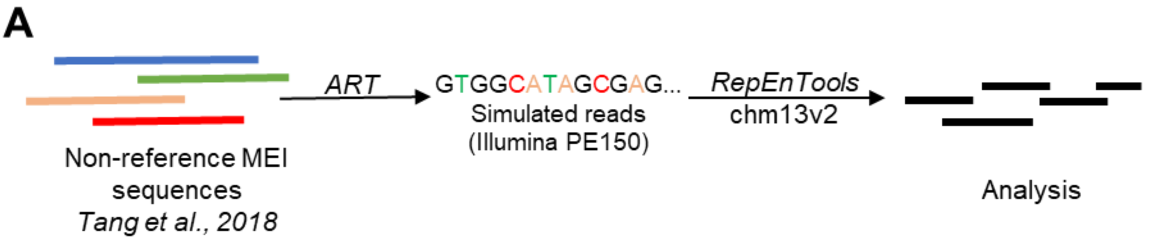

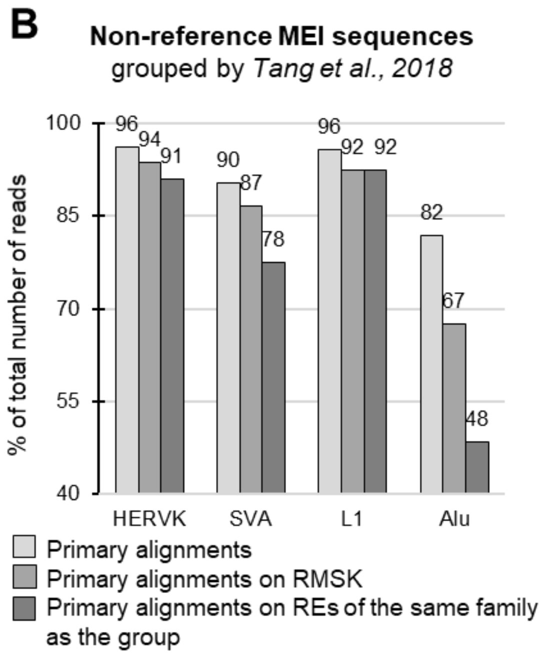

C

	HERVK	SVA	L1	Alu
MEI (n)	10	62	552	1860
Average length (kbp)	4.3	2.8	3.9	1.1
PE reads at 1x simulation (n)	144	584	7213	7248
**CIGAR** mismatches/ MEI kb	5.7	5.4	4.1	4.0
General error rate	0.62%	0.63%	0.44%	0.51%
Reads with ≥ 1 insertions	2.2%	2.3%	0.7%	3.0%
Reads with ≥ 1 deletions	4.0%	3.5%	0.8%	2.8%
Insertions (n)	6	24	98	371
Deletions (n)	11	38	111	336

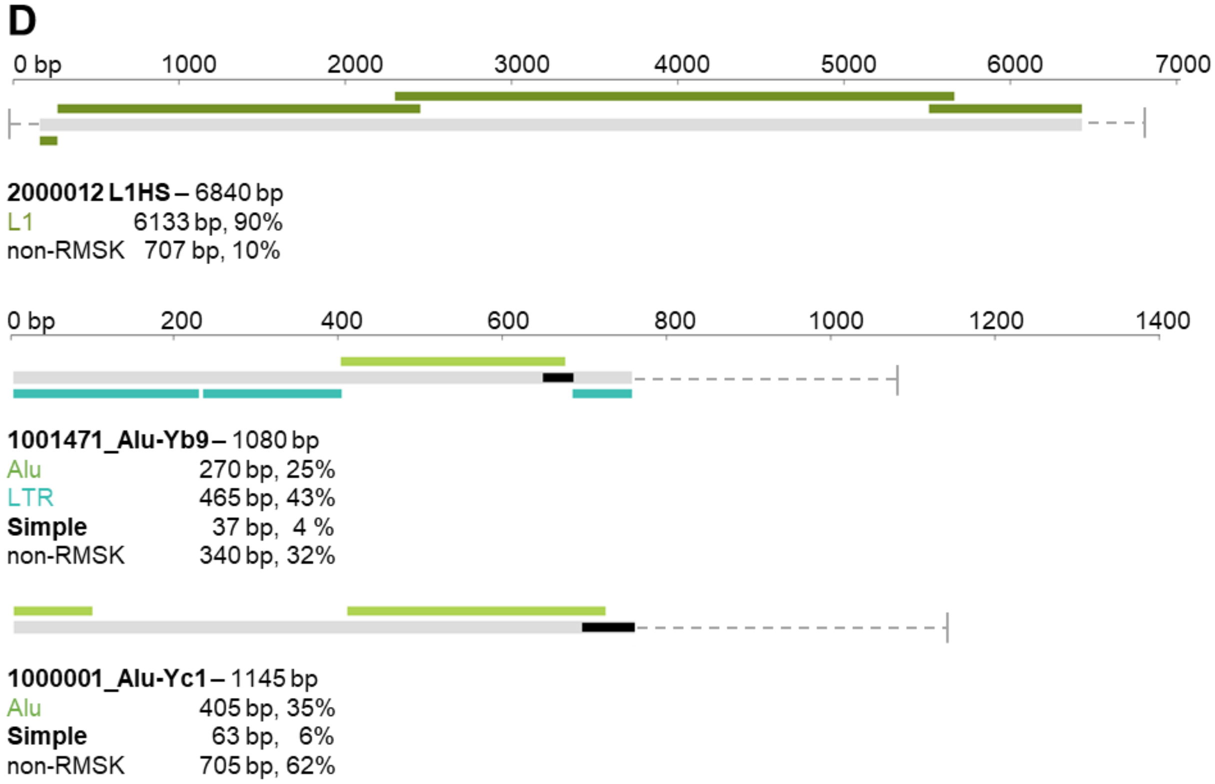

Figure S7. *RepEnTools* analyses REs accurately in simulated data from the mouse mm39 assembly.

A *RepEnTools* demonstrates excellent accuracy in analysis of REs in mm39 as in chm13v2. Plot for mm39 made in equivalent manner to Additional file 1: Fig. S5B. Out of the 18,504 REs in mm39-RMSK, the ones with the lowest read density (first quartile) are essentially exclusively some of the 17,181 Simple repeats. See also Fig. 5C.

B *RepEnTools* accurately analyses REs on mm39 with average relative errors ≤ 1%, even at low coverage. Plot for mm39 made in equivalent manner to Additional file 1: Fig. S5C. Each bar represents the average of n = 1323 REs, excluding the 17,181 Simple repeats, whiskers are the standard error of mean.

C *RepEnTools* demonstrates very accurate data recovery for the young ERVKs (IAP) at a simulation depth resembling typical real experimental data. Plot for mm39 made in equivalent manner to Additional file 1: Fig. S5D.

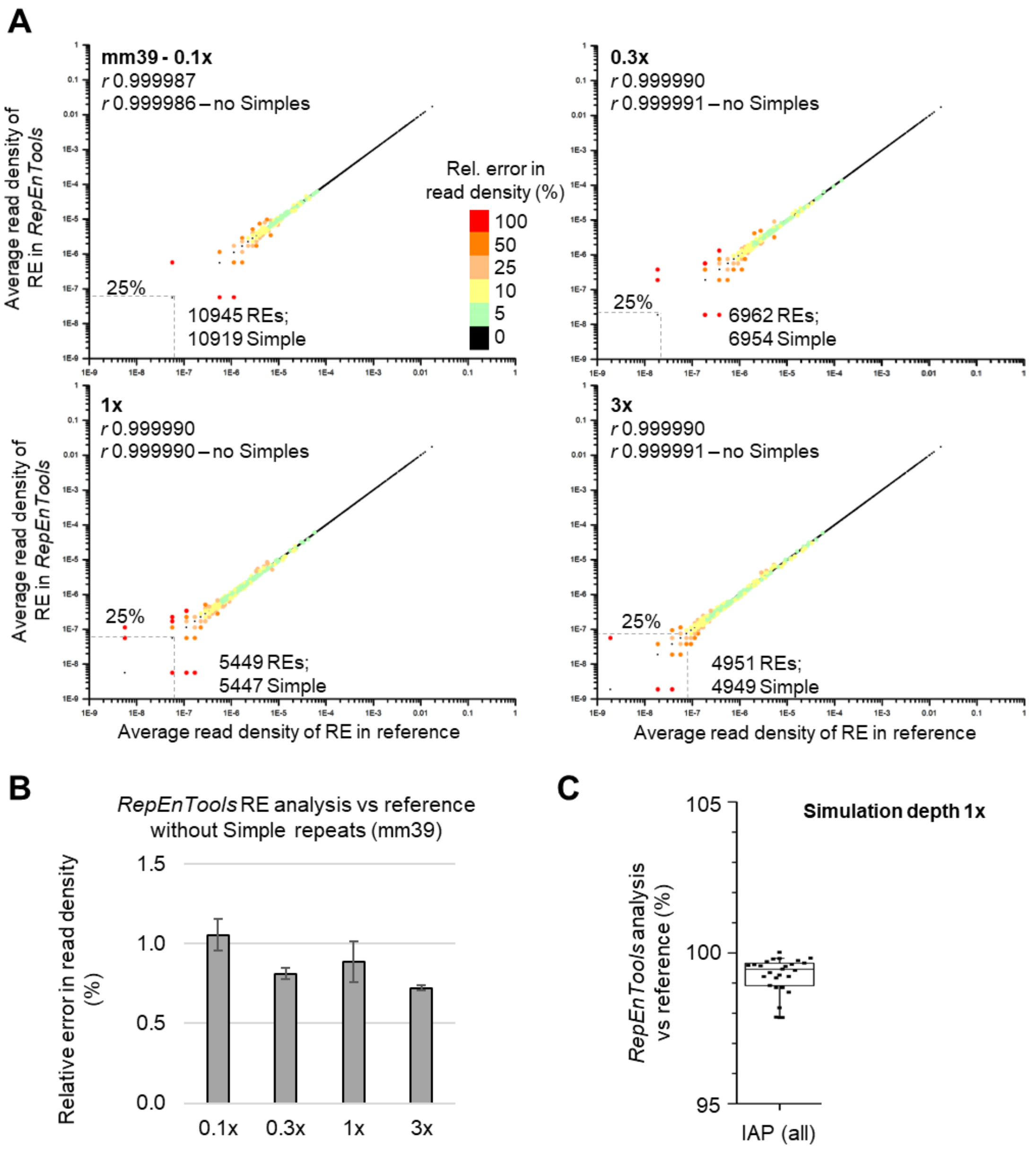

Figure S8. Additional data related to Fig. 6.

A The two replicates of TTD CIDOP have highly correlated (Pearson *r*) signals in 5 kb genome-wide bins, while the input data are only similar to themselves. Data aligned using *RepEnTools-HISAT2*.

B Fingerprint plot demonstrates the enrichment of hUHRF1-TTD CIDOP compared to the input datasets and the reproducibility of both groups. The theoretical perfect input at complete coverage forms the diagonal black line. While the input chromatin data cover a significant part of the genome, the broad TTD curves reflect the broad enrichment peaks observed with this histone modification binding domain (8). Plot generated with *plotFingerprint* (*deepTools2*) (6).

C The majority of hUHRF1-TTD bound regions are on repeat masker (RMSK) annotated regions. The same is true for input. Counts retrieved from *featureCounts* summary using our adjusted RMSK annotation for chm13v2 and compared to primary mapped reads retrieved from *flagstat* for the corresponding file.

D Bar diagram from the *RepEnTools* output showing the ERVK subfamilies least enriched in TTD CIDOP versus input (reproducibility $p \leq 0.05$, $n = 2$). Bar represents the mean, whisker represents the standard deviation. See also Fig. 6C.

E TTD enrichment is found at the 3′ end of essentially all SVA regions that contain this part, confirming the findings of *RepEnTools*. Heatmap of all SVA models (pHMM) on chm13v2, anchored to the 3′ end, and arranged by mean SVA track intensity. SVA track shows position and density of actual SVA annotated segment within the model. pHMM retrieved from RMSK bed12 output found on UCSC Table Browser (1, 2). pHMM – profile Hidden Markov Model. See also Fig. 6D. For an illustration of the differences between pHMM of a RE and the actual RE see Additional file 1: Fig. S2A.

F The TTD peak on the HERVE-int consensus sequence overlaps the promoter and ORF of the pol gene. BLAST analysis matches this to the RNase-H2 domain of a human pol protein (232 of 269 queried aa). The scheme highlights in grey the region of the matching protein sequence. Blast-p performed on blast.ncbi.nlm.nih.gov, domain annotation from InterPro (ebi.ac.uk/interpro/protein) (9). See also Fig. 6G, Additional file 2: Text S2, and Fig. S9B.

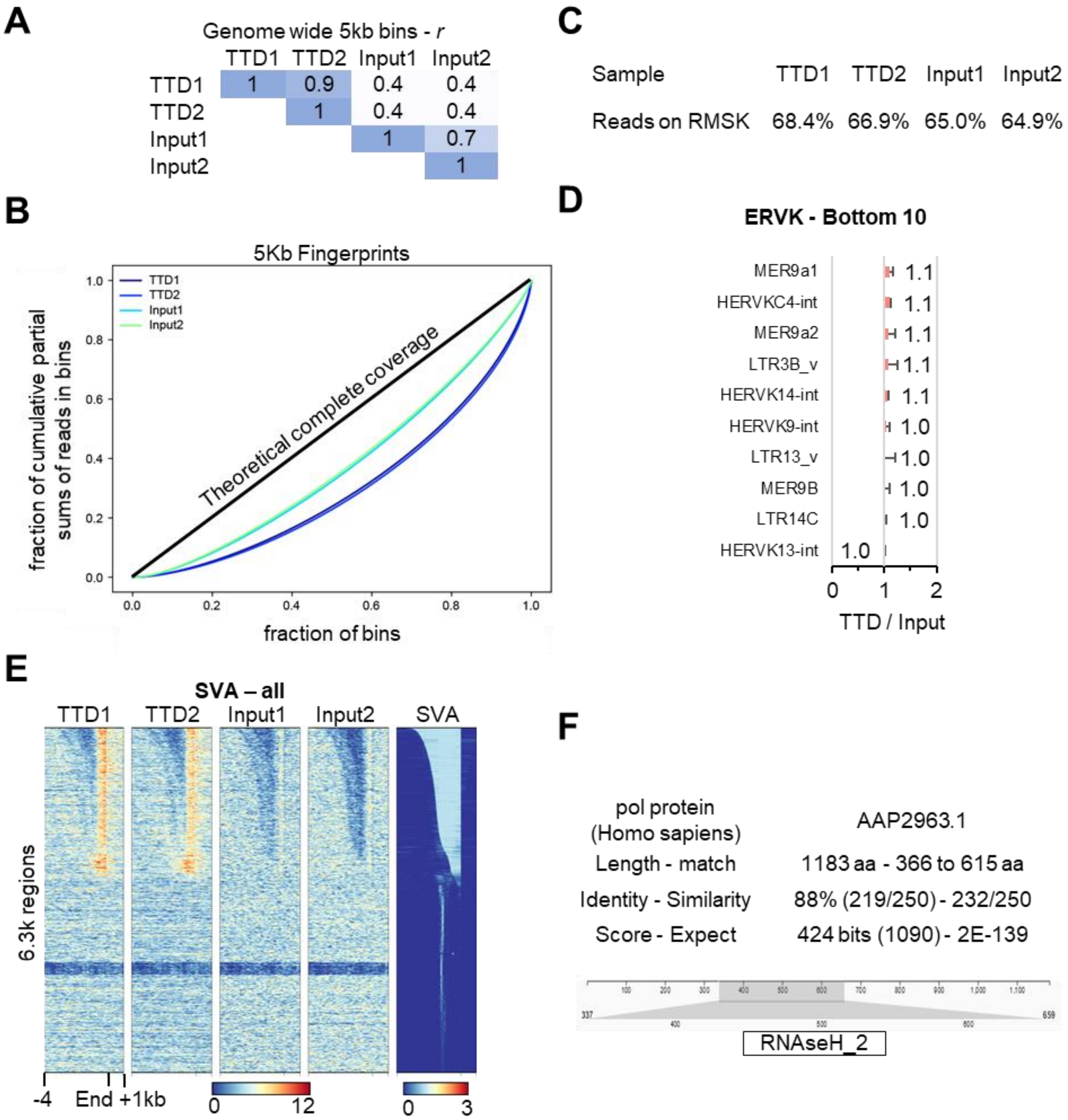
A
Genome wide 5kb bins - r
TTD1 TTD2 Input1 Input2
TTD1 1 0.9 0.4 0.4
TTD2 1 0.4 0.4
Input1 1 0.7
Input2 1
B
5Kb Fingerprints
TTD1
TTD2
Input1
Input2
Theoretical complete coverage
fraction of cumulative partial sums of reads in bins
fraction of bins
C
Sample TTD1 TTD2 Input1 Input2
Reads on RMSK 68.4% 66.9% 65.0% 64.9%
D
ERVK - Bottom 10
MER9a1 1.1
HERVKC4-int 1.1
MER9a2 1.1
LTR3B_v 1.1
HERVK14-int 1.1
HERVK9-int 1.0
LTR13_v 1.0
MER9B 1.0
LTR14C 1.0
HERVK13-int 1.0
TTD / Input
E
SVA – all
TTD1 TTD2 Input1 Input2 SVA
6.3k regions
-4 End +1kb
0 12
0 3
F
pol protein (Homo sapiens) AAP2963.1
Length - match 1183 aa - 366 to 615 aa
Identity - Similarity 88% (219/250) - 232/250
Score - Expect 424 bits (1090) - 2E-139
RNAseH_2

Figure S9. Design of qPCR assays for *RepEnTools* corroboration.

A The depicted workflow was used to design and validate the qPCR assays developed in this study to corroborate enrichment and depletion results of *RepEnTools*. PCR amplification of REs in a quantitative manner is non-trivial, making careful validation of the assays critical. A previous report of qPCR assays targeting the human alpha satellites made use of a similar validation scheme (10). Dfam (11), *in-silico PCR-BLAT* (genome.ucsc.edu/cgi-bin/hgPcr) (12) and *Primer blast* (ncbi.nlm.nih.gov/tools/primer-blast/)(13) were important tools for primer design. The selection criteria used here are stringent and in line with the best-practice recommendations in the literature (14).

B Schemes of the amplicon positions on the dfam consensi of significantly enriched and depleted REs in hUHRF1-TTD CIDOP according to *RepEnTools*. Dfam names, codes and sequence length are indicated in the top left. Functional elements and approximate TTD enrichment peaks are indicated as boxes. See also Fig. 6G.

C Amplicon length according to design and predicted number of perfect matches to the primers according to *in-silico PCR-BLAT*. The control regions (H3K9me2, H3K4me3) targeting single genomic loci were used previously (8). The negative controls had zero perfect matches, and *Primer-BLAST* results of plausible matches with < 150 bp amplicon length are listed instead. All primer pairs designed using the consensi of REs have multiple targets of essentially identical amplicon sizes. This recaptures the spirit of *RepEnTools* analysis, consolidating similar RE sequences, regardless of specific genomic location or polymorphic character.

D Amplification programs used for the primer pairs described in this study. The qPCR assays were performed on a CFX96 qPCR system (Bio-Rad) using the ORASEE qPCR reagent (highQu). The oligonucleotides used for qPCR assays are listed in Additional file 3: Table S2. We achieved optimal results for REs by following the recommendation for fast, two-step protocols (15).

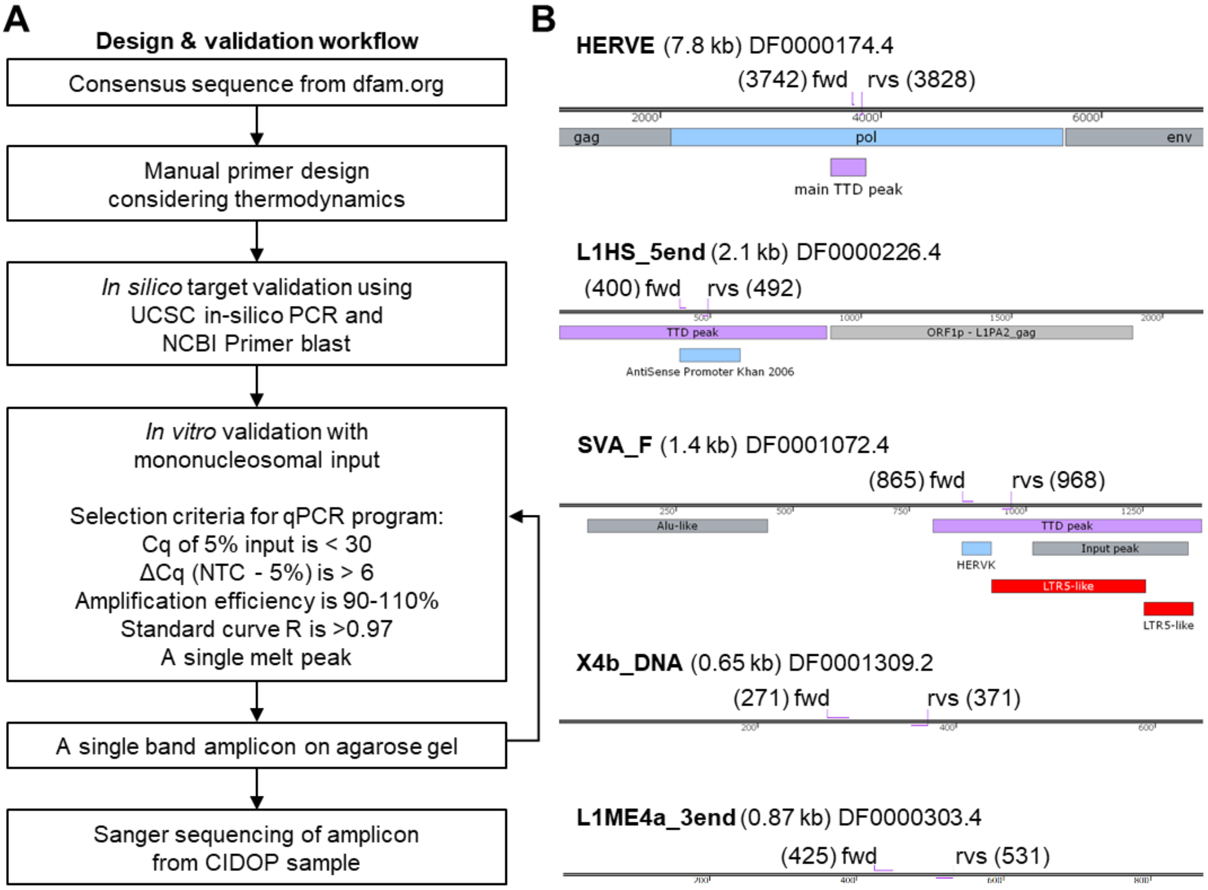

C

Name	Amplicon length (bp)	Number of target sites	chm13v2 coordinates
H3K9me2	89	1	chr7:105522367-105522475
H3K4me3	109	1	chr3:196409931-196410019
HERVE	87	38	
L1HS	93	670	
SVA-F	104	316	
X4b	101	3 (Primer-BLAST)	
L1ME4a	107	21 (Primer-BLAST)	

D

H3K9me2	H3K4me3	HERVE	L1HS	SVA-F	X4b	L1ME4a	
95°C (3min)	95°C (3min)	95°C (3min)	95°C (3min)	95°C (3min)	95°C (3min)	95°C (3min)	
95°C (5s)	95°C (5s)	95°C (5s)	95°C (5s)	95°C (5s)	95°C (5s)	95°C (5s)	x40 cycles
58°C (10s)	58°C (10s)						
72°C (10s)	72°C (10s)	68°C (15s)	67°C (15s)	70°C (15s)	60°C (5s)	56°C (10s)	

Figure S10. Validation of qPCR assays for *RepEnTools* corroboration.

The assays developed here should be useful to other researchers, once validated for the materials available in-house and the cell-line of interest.

A All qPCR assays (primer pairs and corresponding amplification programs) presented here fulfil the stringent selection criteria outlined in Fig. S9A, in line with the literature (14). Amplification efficiencies are consistently approaching 100%, the upper calibration points (5%) are within appropriate range, and unspecific signal from non-template controls (NTC) is at least $2^{11.4-1}$ = 1,351 times lower. The assays were validated using HepG2 mononucleosomal input, for $n \geq 3$ biological replicates. For each primer pair, a 3-point quantitation curve was generated using serial dilutions of the input (5%, 1%, 0.2%) and NTC as negative controls. The quantitation model used was the "single-threshold with serial dilution calibration and efficiency correction". Amplification efficiencies, quantification cycle (Cq) etc were retrieved from *CFX Maestro* v2.3 (Bio-Rad). This report addresses the major essential information for non-RT qPCR in the spirit of the MIQE guidelines (16, 17).

B Exemplary melt curves from RE targeting assays demonstrate that the amplified sequences are consistent throughout the calibration range. The data in each plot are from a single biological replicate and all the wells that make up a complete 3-point quantitation curve, covering the entire calibration range.

C Exemplary agarose gels of the 5% input and TTD WT CIDOP-qPCR products from RE targeting assays demonstrate the suitability of the assays for sample quantitation, given the amplification of single band products of similar size for both sample types.

D Sanger sequencing of TTD WT CIDOP-qPCR products confirmed the selective amplification of sequences from the same type of RE. The targets contain the same sequence as the two primers on the flanks but are otherwise diverse, demonstrating the multiplicity of the amplicons. Query of the Sanger sequences on dfam.org returned a unique result of the corresponding targeted RE, verifying that the sequences detected from the amplicons are specific to this RE.

E TTD CIDOP-qPCR for WT and the binding deficient D142A mutant in both biological replicates reproduced the enrichment and depletion reported in our *RepEnTools* analysis, using the carefully validated qPCR assays we developed for this study. Design and validation of qPCR assays is particularly troublesome when the target REs have small tandem repeat sequences (e.g. GSAT, ACRO, SATR1, SATR2), very AT-rich nature (e.g. DNA1_Mam), and when other challenges are encountered (MNase digested amplicons, great divergence from consensus sequence, very low abundance etc).

A

	HERVE	L1HS	SVA-F	X4b	L1ME4a
Mean efficiency (±SD)	99.6 % (±1.3)	94.4 % (±2.9)	97.9 % (±1.8)	96.5 % (±3.6)	91.8 % (±8.9)
Mean Cq of 5% input (±SD)	21.9 (± 0.2)	17.4 (± 0.2)	19.4 (± 0.2)	28.8 (± 0.2)	29.3 (±0.2)
Δ Cq (NTC - 5%)	16.4	12.8	13.9	12.2	11.4

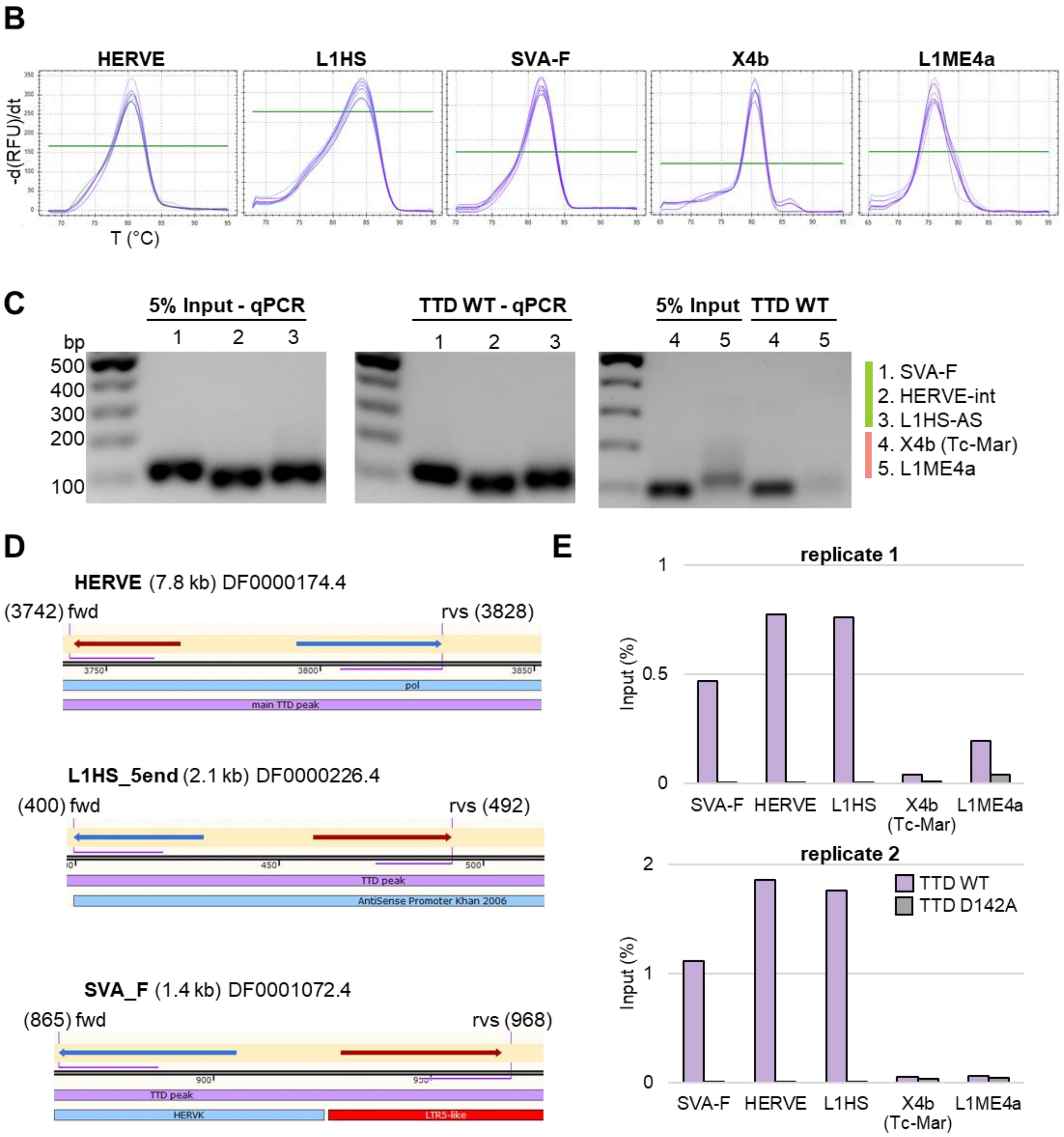

Figure S11. Control data related to Fig. 7.

A *RepEnTools* found significant enrichment in the L1PBa1 subfamily (Fig. 7A), where we see reproducible enrichment of TTD over Input at the 5′ end of the elements. Profile of all L1PBa1 models (pHMM) (331 regions), anchored to the 5′ end. RE track shows the density of actual L1PBa1 annotated segments within the model. RE annotation from dfam consensus sequence (11). pHMM – profile Hidden Markov Model.

B Some TTD depletion is found at the 3′ end of most L1ME regions that contain it. Heatmap of all L1ME models, anchored to the 5′ end. L1ME track shows position and density of actual L1ME annotated segments within the model. See also Fig. 7B.

C Evolutionary younger L1PA subfamilies contain more full-length elements than older ones, in chm13v2. The bar diagram shows the ratio of the subfamily frequency among full-length L1PAs versus among L1PA pHMM models.

D hUHRF1-TTD CIDOP enrichment does not correlate with the simple abundance of L1PA elements. Only reproducibly enriched/depleted subfamilies are annotated ($p \leq 0.05$, $n = 2$). See also Fig. 7D.

E Localised TTD enrichment is found at the 5′ UTR of the full-length L1PAs, and at the intergenic region before ORF2. Profile of all L1PA models (8,295 regions), anchored to the 5′ end. RE track shows the density of actual L1PA annotated segments within the model. Annotations compiled using information from dfam (5′ UTR, ORF1), and specialised reports (11, 18, 19).

F Control data show that there is no TTD enrichment over full-length L1MEs. Profile of full-length L1MEs (512 regions).

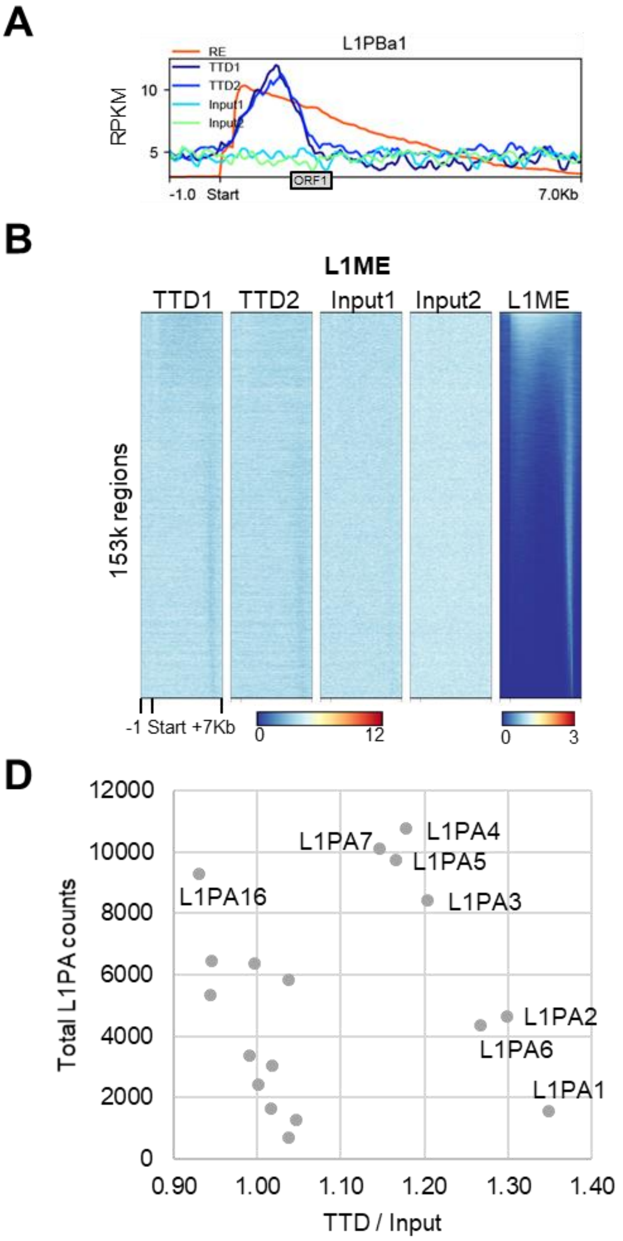
A
L1PBa1
RE
TTD1
TTD2
Input1
Input2
RPKM
10
5
-1.0 Start
ORF1
7.0Kb
B
L1ME
TTD1
TTD2
Input1
Input2
L1ME
153k regions
-1 Start +7Kb
0
12
0
3
C
Full-length / total
L1PA frequencies in chm13v2
0
1
2
3
L1PA1
L1PA2
L1PA3
L1PA4
L1PA5
L1PA6
L1PA7
L1PA8
L1PA8A
L1PA10
L1PA11
L1PA12
L1PA13
L1PA14
L1PA15
L1PA15-16
L1PA16
L1PA17
D
Total L1PA counts
12000
10000
8000
6000
4000
2000
0
0.90
1.00
1.10
1.20
1.30
1.40
TTD / Input
L1PA7
L1PA4
L1PA5
L1PA16
L1PA3
L1PA2
L1PA6
L1PA1
E
L1PA (full-length)
RE
TTD1
TTD2
Input1
Input2
RPKM
10
5
-1.0 Start
5'
ORF1
ORF2
3'
7.0Kb
F
L1ME (full-length)
RE
TTD1
TTD2
Input1
Input2
RPKM
10
5
-1.0 Start
7.0Kb

Figure S12. hUHRF1-TTD binds to H3K4me1-K9me3 on functional enhancers in L1PAs.

A TTD enrichment at the 5′ end of most full-length L1PAs overlaps with H3K4me1 and H3K9me3 signals. Otherwise, TTD peak signal occurs on broad H3K9me2 co-occurring with H3K4me1, as we described in detail previously (8). Heatmap of full-length L1PAs, anchored to the 5′ end, and arranged by RE length. TTD and H3K9me2 from Choudalakis et al., 2023. H3K4me1 and H3K9me3 (marked with *) from Arrigoni et al., 2018 (20). The change in scale reflects the differences in experimental and sequencing methods. See also Fig. 7C.

B Motif analysis of TTD enriched sequences versus input from full-length L1PA1/L1HS regions reveals reproducible and statistically significant enrichment in transcription factor binding motifs corresponding to KLF4 and E2F4. Analysis was performed on *XSTREME – MEME suite* (21) using the aligned sequences from TTD CIDOP extracted as FASTA. Controls were the input sequences from the same regions, as each biological replicate was compared to its respective input. See also Fig. 7F.

C TTD enrichment at the 5′ end of most full-length SVAs overlaps with H3K4me1 and H3K9me3 signals. TTD enrichment colocalises with the H3K4me1-K9me2/3 double marks. This overlaps the HERVK and LTR5-like regions of full-length SVAs, shown to harbour TE Enhancers (1, 22). See also Fig. 7G.

D TTD signal is strongest where H3K4me1 colocalises with H3K9me3, forming the H3K4me1-K9me3 double mark on and around full-length LTR22s. H3K4me1 and H3K9me3 ChIP-seq RPKM scale on the right axis. RE track shows density of actual LTR22 annotated segments within the model. Profile of all full-length LTR22s (599 regions).

E TTD signal is overlapping the H3K4me1-K9me2 double mark on and around the HERVE annotated genomic loci. Profile of all HERVE models (142 regions). See also Fig. 6F.

F Sequences from the REs of interest can have enhancer function in HepG2 cells. STARR-peaker peaks for HepG2 cells (23) were retrieved from ENCODE (ENCFF047LDJ), lifted to chm13v2 and overlapped with the RMSK annotation. As HERVE annotated sequences are typically truncated (97.8%), the RE model coordinates were used here to avoid underreported overlap due to minimal region size.

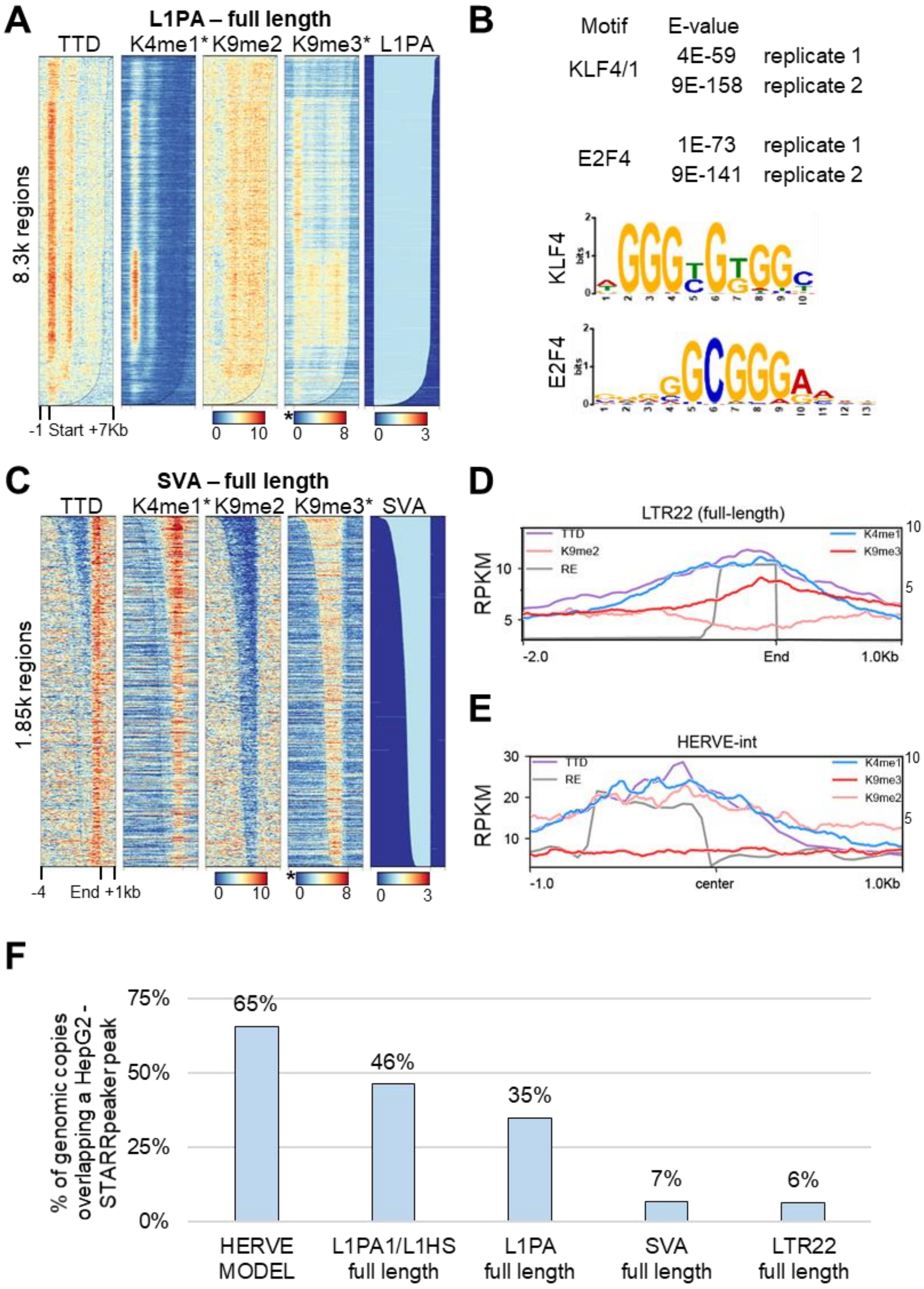
A
L1PA – full length
TTD
K4me1*
K9me2
K9me3*
L1PA
8.3k regions
-1 Start +7Kb
0 10
0 8
0 3
B
Motif
E-value
KLF4/1
4E-59
replicate 1
9E-158
replicate 2
E2F4
1E-73
replicate 1
9E-141
replicate 2
KLF4
E2F4
C
SVA – full length
TTD
K4me1*
K9me2
K9me3*
SVA
1.85k regions
-4 End +1kb
0 10
0 8
0 3
D
LTR22 (full-length)
TTD
K9me2
RE
K4me1
K9me3
RPKM
-2.0
End
1.0Kb
E
HERVE-int
TTD
RE
K4me1
K9me3
K9me2
RPKM
-1.0
center
1.0Kb
F
% of genomic copies overlapping a HepG2 - STARRpeaker peak
75%
50%
25%
0%
65%
46%
35%
7%
6%
HERVE MODEL
L1PA1/L1HS full length
L1PA full length
SVA full length
LTR22 full length

Figure S13. Visualisations of the reproducibility of *RepEnTools* alignments on L1PA *loci*.

A Reads are well mapped and reproducibly aligned as visualised on specific instances of L1PA *loci* by *RepEnTools*. At the 5′ end of a representative full-length L1PA1, the enrichment is experimentally and bioinformatically well reproduced in two biological replicates of hUHRF1-TTD CIDOP processed by *RepEnTools*. Good mappability is shown using simulated reads mapped by *RepEnTools*. These are very similar to the reference fragments generated by ART for sequencing depths 1x and 3x.

B A second representative browser view supports the same conclusions for the 5′ end of a representative full-length L1PA2. Sample tracks in RPKM, y-axes start from 0. Coordinates in chm13v2. Panels made with IGV (software.broadinstitute.org/software/igv/).

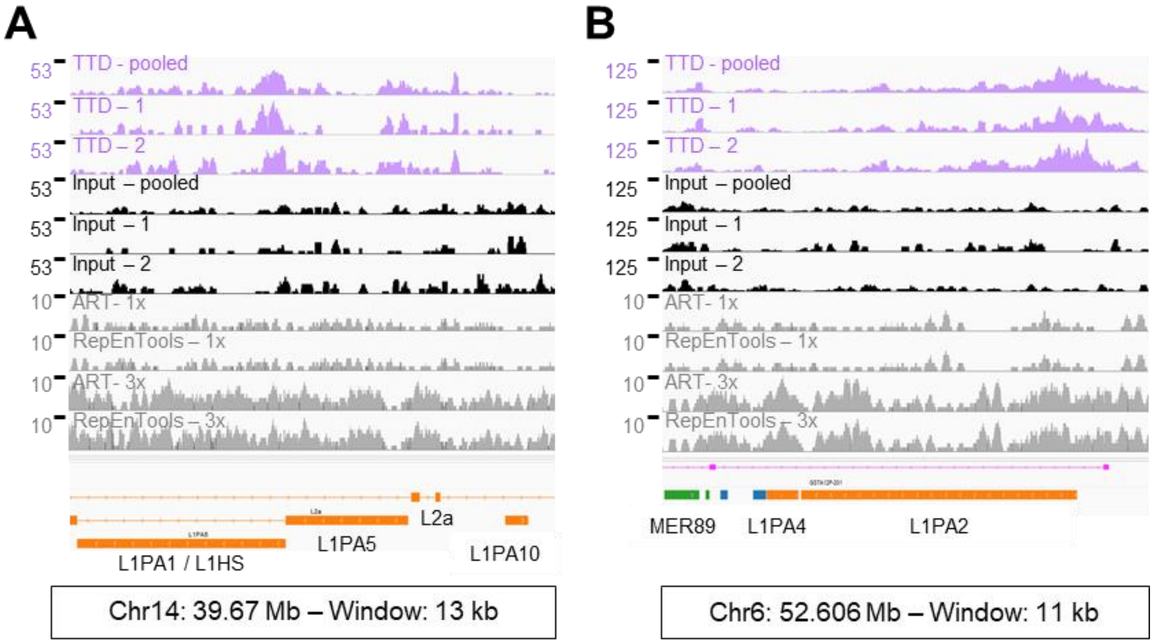

Supplementary references

1. Hoyt SJ, Storer JM, Hartley GA, Grady PGS, Gershman A, de Lima LG, et al. From telomere to telomere: The transcriptional and epigenetic state of human repeat elements. Science. 2022;376(6588):eabk3112.
2. Karolchik D, Hinrichs AS, Furey TS, Roskin KM, Sugnet CW, Haussler D, et al. The UCSC Table Browser data retrieval tool. Nucleic Acids Res. 2004;32(Database issue):D493-6.
3. Thorvaldsdottir H, Robinson JT, Mesirov JP. Integrative Genomics Viewer (IGV): high-performance genomics data visualization and exploration. Brief Bioinform. 2013;14(2):178-92.
4. Teissandier A, Servant N, Barillot E, Bourc'his D. Tools and best practices for retrotransposon analysis using high-throughput sequencing data. Mob DNA. 2019;10:52.
5. Dickson BM, Kupai A, Vaughan RM, Rothbart SB. Streamlined quantitative analysis of histone modification abundance at nucleosome-scale resolution with siQ-ChIP version 2.0. Sci Rep. 2023;13(1):7508.
6. Ramirez F, Ryan DP, Gruning B, Bhardwaj V, Kilpert F, Richter AS, et al. deepTools2: a next generation web server for deep-sequencing data analysis. Nucleic Acids Res. 2016;44(W1):W160-5.
7. Tang W, Mun S, Joshi A, Han K, Liang P. Mobile elements contribute to the uniqueness of human genome with 15,000 human-specific insertions and 14 Mbp sequence increase. DNA Res. 2018;25(5):521-33.
8. Choudalakis M, Kungulovski G, Mauser R, Bashtrykov P, Jeltsch A. Refined read-out: The hUHRF1 Tandem-Tudor domain prefers binding to histone H3 tails containing K4me1 in the context of H3K9me2/3. Protein Sci. 2023;32(9):e4760.
9. Paysan-Lafosse T, Blum M, Chuguransky S, Grego T, Pinto BL, Salazar GA, et al. InterPro in 2022. Nucleic Acids Res. 2023;51(D1):D418-D27.
10. Contreras-Galindo R, Fischer S, Saha AK, Lundy JD, Cervantes PW, Mourad M, et al. Rapid molecular assays to study human centromere genomics. Genome Res. 2017;27(12):2040-9.
11. Storer J, Hubley R, Rosen J, Wheeler TJ, Smit AF. The Dfam community resource of transposable element families, sequence models, and genome annotations. Mob DNA. 2021;12(1):2.
12. Kent WJ. BLAT--the BLAST-like alignment tool. Genome Res. 2002;12(4):656-64.
13. Ye J, Coulouris G, Zaretskaya I, Cutcutache I, Rozen S, Madden TL. Primer-BLAST: a tool to design target-specific primers for polymerase chain reaction. BMC Bioinformatics. 2012;13:134.
14. Bustin S, Huggett J. qPCR primer design revisited. Biomol Detect Quantif. 2017;14:19-28.
15. Bustin SA. How to speed up the polymerase chain reaction. Biomol Detect Quantif. 2017;12:10-4.

16. Bustin SA, Benes V, Garson J, Hellemans J, Huggett J, Kubista M, et al. The need for transparency and good practices in the qPCR literature. Nat Methods. 2013;10(11):1063-7.

17. Bustin SA, Benes V, Garson JA, Hellemans J, Huggett J, Kubista M, et al. The MIQE guidelines: minimum information for publication of quantitative real-time PCR experiments. Clin Chem. 2009;55(4):611-22.

18. Khan H, Smit A, Boissinot S. Molecular evolution and tempo of amplification of human LINE-1 retrotransposons since the origin of primates. Genome Res. 2006;16(1):78-87.

19. Boissinot S, Sookdeo A. The Evolution of LINE-1 in Vertebrates. Genome Biol Evol. 2016;8(12):3485-507.

20. Arrigoni L, Al-Hasani H, Ramirez F, Panzeri I, Ryan DP, Santacruz D, et al. RELACS nuclei barcoding enables high-throughput ChIP-seq. Commun Biol. 2018;1:214.

21. Grant CE, Bailey TL. XSTREME: Comprehensive motif analysis of biological sequence datasets. bioRxiv. 2021:2021.09.02.458722.

22. Pontis J, Planet E, Offner S, Turelli P, Duc J, Coudray A, et al. Hominoid-Specific Transposable Elements and KZFPs Facilitate Human Embryonic Genome Activation and Control Transcription in Naive Human ESCs. Cell Stem Cell. 2019;24(5):724-35 e5.

23. Lee D, Shi M, Moran J, Wall M, Zhang J, Liu J, et al. STARRPeaker: uniform processing and accurate identification of STARR-seq active regions. Genome Biol. 2020;21(1):298.
