# Supplementary material for: RepEnTools: an automated repeat enrichment analysis package for ChIP-seq data reveals hUHRF1 Tandem-Tudor domain enrichment in young repeats
Source: Mob DNA. 2024 Apr 3;15:6. doi: 10.1186/s13100-024-00315-y (PMC10988844; doi:10.1186/s13100-024-00315-y)
Supplement: Supplementary file 2 — Additional file 2. Supplementary Texts S1-2. [file 13100_2024_315_MOESM2_ESM.pdf]

RepEnTools: An automated repeat enrichment analysis package for ChIP-seq data reveals hUHRF1 Tandem-Tudor domain enrichment in young repeats

Michel Choudalakis, Pavel Bashtrykov* & Albert Jeltsch*

Additional file 2

Supplementary Text

Text S1. Definitions of terminology used in this study.

Some terms have been used in unclear or non-specific manner in the literature. For clarity we define our usage of the terms, in line with the SAM specifications (v44b4167_22/Aug/22) (github.com/samtools/hts-specs).

- Fragment: a fragmented dsDNA molecule.
- Read: a sequenced, not inferred, string of DNA data.
- PE read: paired-end reads. This number is used in sequencing depth estimates and essentially corresponds to the number of reads divided by two. It is sensible for comparisons to single-end experiments.
- Insert: a sequenced or inferred string of mapped DNA sequence delimited by the ends of mapped paired reads.
- Alignment: one set of coordinates for a single read, a read alignment per SAM specifications.
- Multi-mapping read: a read with multiple ambiguous alignments of equal quality.
- Primary alignment: a read alignment that is neither secondary nor supplementary (eq. flags 256 and 2048). In *HISAT2*, there is only one primary alignment for every mapped read. Specifically, for multi-mapping reads, one alignment is randomly assigned the primary role and all others are secondary.
- Alignment algorithm: alignment software with specific, consistent settings

Repeat element names and naming conventions are according to RMSK (chm13v2), as *RepEnTools* is based on it, or Dfam for consensi. The two are the extensively overlapping. We acknowledge the existence of various classification schemes.
For clarity we provide examples to the terms we use:
Repeat element class e.g. LTR or LINE
Repeat element superfamily e.g. ERV or L1
Repeat element family e.g. ERVK or L1PA
Repeat element subfamily or repeat element e.g. HERVK-9 or L1PA1
Repeat element instance e.g. HERVK-9 or L1PA1 at specific coordinates

Text S2. Sequence of the ORF from the HERVE consensus pol gene that is overlapped by the main TTD peak (269 aa, 29.8 kDa).

MPY(S/P/T/A)GTWRTVGIRCPRR(N/K)AQICRRQVRYLGFTIRQGERSPGSERKQVICN
LPEPKSRRQVREFLGAVGFCRLWIPNFAVLAKPLYEVTKGGDREPLEWGSQQQQVFH
ELKEKLLAAPALGLPDLTKPFPLYASEREKMAAGLLTQTVGPWPRPVAYLSKQLDGV
SKGWPPCLRALAATALLVQEANKLTLGQNLNIKAPHAVVTLMNTKGHHWLTNARL
TKYQTLLCENPRITIEVCNTLHPATLLPVSESPVE(L/P/H/R)DCVEVLDSVDSGHQ*

Ambiguities are due to variable nucleotides in the consensus sequence.
