# Supplementary material for: RepEnTools: an automated repeat enrichment analysis package for ChIP-seq data reveals hUHRF1 Tandem-Tudor domain enrichment in young repeats
Source: Mob DNA. 2024 Apr 3;15:6. doi: 10.1186/s13100-024-00315-y (PMC10988844; doi:10.1186/s13100-024-00315-y)
Supplement: Supplementary file 3 — Additional file 3. Supplementary Tables S1-3. [file 13100_2024_315_MOESM3_ESM.pdf]

RepEnTools: An automated repeat enrichment analysis package for ChIP-seq data reveals hUHRF1 Tandem-Tudor domain enrichment in young repeats

Michel Choudalakis, Pavel Bashtrykov* & Albert Jeltsch*

Additional file 3

Supplementary Tables

Table S1. Approximate evolutionary age of selected repeat elements.

Table S2. Oligonucleotides used for qPCR assays in this study.

Table S3. NGS public datasets used in this study.

Supplementary references

Supplementary Tables

Table S1. Approximate evolutionary age of selected repeat elements.

RE class	Element	Approximate age (Myrs)
**Retroposons**	SVA-F, SVA-E	3
	SVA-D, SVA-C	9.5-11
	SVA-B, SVA-A	11.6-13.6
**LINE-1**	L1HS/L1PA1	3
	L1PA2-3, L1P1	8-13
	L1PA4-6, L1P2	18-27
	L1PA7-8A, L1P3	31-42
	L1PA10-17, L1P4	46-101
	L1PBa1-a, L1P	~63
	L1MA	62-102
	L1ME	>100
**LTRs**	ERVK	0.5-55
	ERV1/H	8-<100
	ERVL	>100
	ERVL-MaLR	>100
	Gypsy-LTR	>100

Evolutionary age for repeat elements of interest in this study, arranged from youngest to oldest. Data retrieved from Wang et al. 2005 (SVA), Khan et al. 2006 (L1PA), Giordano et al. 2007 (other LINE-1s) (1-3). Long terminal repeat (LTR) ages are compiled from (4), and ordered as previously (5). The broad ranges among LTRs are due to different waves of retrotranspositional amplification bursts (4). The interested reader is cautioned regarding the different naming and classification schemes for LTRs among the various sources in the literature and the databases, e.g. LTR22, LTR22A, and LTR22B are collectively named HERVK22I or HERV-K(HML-5) or NMWV2 (4).

Table S2. Oligonucleotides used for qPCR assays in this study.

Name	Forward primer	Reverse primer	Amplicon length (bp)
H3K9me2	atgattatgagcccaccaggc	agagtcagcctttgatgcca	89
H3K4me3	actctcttctcgctggtcct	tccatgtcgtctccttagcc	109
HERVE	ccctgtttgagggccttggc	ggcctttatgttcaggttttgccc	87
L1HS	ctgagatcaaactgcaaggcgg	gagcttcccggctgctttg	93
SVA-F	cagctcattgagaacgggccagg	cacagacacggcaaccatccg	104
X4b	ctgccatcaagtagcaattacc	tgggtggtttgggagtt	101
L1ME4a	acaaagatttatgtacaaggatgttc	ccataatttatttaaccaCtcccc	107

Primers were manufactured by IDT as 25 nmole DNA oligonucleotides, with standard desalting as purification. No modifications were introduced.

Table S3. NGS public datasets used in this study.

Cells	Name	GEO code	Affinity reagent	Ref.
HepG2	hUHRF1-TTD	GSM6593324-5	hUHRF1-TTD (residues 126-280)	(6)
	H3K9me2	GSM6593326-7	abcam, ab1220, lot GR3377057-1	
	Input	GSM6593328-9	n.a.	
	H3K4me1	GSM3019940	Diagenode, C15410194, lot A1863-001D	(7)
	H3K9me3	GSM3019942	Diagenode, C15410193, lot A1671-001P	
	Input	GSM3019946	n.a.	
mESC	mUHRF1	GSM4801078-9	α-FLAG	(8)
	input	GSM4801090-1	n.a.	
	H3K9me2	GSM4672092	abcam, ab32521	(9)
	H3K4me1	GSM5233312 -3	abcam, ab8895	(10)
	H3K9me3	GSM5233300-1	abcam, ab8898	

Published ChIP-seq data were downloaded as fastq files.

Supplementary references

1. Wang H, Xing J, Grover D, Hedges DJ, Han K, Walker JA, et al. SVA elements: a hominid-specific retroposon family. J Mol Biol. 2005;354(4):994-1007.
2. Giordano J, Ge Y, Gelfand Y, Abrusan G, Benson G, Warburton PE. Evolutionary history of mammalian transposons determined by genome-wide defragmentation. PLoS Comput Biol. 2007;3(7):e137.
3. Khan H, Smit A, Boissinot S. Molecular evolution and tempo of amplification of human LINE-1 retrotransposons since the origin of primates. Genome Res. 2006;16(1):78-87.
4. Bannert N, Kurth R. The evolutionary dynamics of human endogenous retroviral families. Annu Rev Genomics Hum Genet. 2006;7:149-73.
5. Hoyt SJ, Storer JM, Hartley GA, Grady PGS, Gershman A, de Lima LG, et al. From telomere to telomere: The transcriptional and epigenetic state of human repeat elements. Science. 2022;376(6588):eabk3112.
6. Choudalakis M, Kungulovski G, Mauser R, Bashtrykov P, Jeltsch A. Refined read-out: The hUHRF1 Tandem-Tudor domain prefers binding to histone H3 tails containing K4me1 in the context of H3K9me2/3. Protein Sci. 2023;32(9):e4760.
7. Arrigoni L, Al-Hasani H, Ramirez F, Panzeri I, Ryan DP, Santacruz D, et al. RELACS nuclei barcoding enables high-throughput ChIP-seq. Commun Biol. 2018;1:214.
8. Haggerty C, Kretzmer H, Riemenschneider C, Kumar AS, Mattei AL, Bailly N, et al. Dnmt1 has de novo activity targeted to transposable elements. Nat Struct Mol Biol. 2021;28(7):594-603.
9. Fu H, Zhang W, Li N, Yang J, Ye X, Tian C, et al. Elevated retrotransposon activity and genomic instability in primed pluripotent stem cells. Genome Biol. 2021;22(1):201.
10. Barral A, Pozo G, Ducrot L, Papadopoulos GL, Sauzet S, Oldfield AJ, et al. SETDB1/NSD-dependent H3K9me3/H3K36me3 dual heterochromatin maintains gene expression profiles by bookmarking poised enhancers. Mol Cell. 2022;82(4):816-32 e12.
